# Antenna-enhanced mid-infrared detection of extracellular vesicles derived from human cancer cell cultures

**DOI:** 10.1186/s12951-022-01693-2

**Published:** 2022-12-13

**Authors:** Maria Eleonora Temperini, Flavio Di Giacinto, Sabrina Romanò, Riccardo Di Santo, Alberto Augello, Raffaella Polito, Leonetta Baldassarre, Valeria Giliberti, Massimiliano Papi, Umberto Basile, Benedetta Niccolini, Ewa K. Krasnowska, Annalucia Serafino, Marco De Spirito, Alessandra Di Gaspare, Michele Ortolani, Gabriele Ciasca

**Affiliations:** 1 Department of Physics, Sapienza University of Rome, Piazzale Aldo Moro 2, 00185 Rome, Italy; 2 Center for Life Neuro and Nano Sciences IIT@Sapienza, Istituto Italiano di Tecnologia, Viale Regina Elena 291, 00161 Rome, Italy; 3 Fondazione Policlinico Universitario “A. Gemelli”, IRCCS, Rome, Italy; 4 Dipartimento di Neuroscienze, Sezione di Fisica, Università Cattolica del Sacro Cuore, Rome, Italy; 5 Dipartimento di Scienze di Laboratorio e Infettivologiche, Fondazione Policlinico Universitario “A. Gemelli” IRCCS, 00168 Rome, Italy; 6 Institute of Translational Pharmacology, National Research Council of Italy, Rome, Italy; 7 NEST, CNR-Istituto Nanoscienze and Scuola Normale Superiore, Piazza San Silvestro 12, 56127 Pisa, Italy

**Keywords:** Biosensors, Plasmonics, SEIRA, IR spectroscopy, Extracellular Vesicles, Nanomaterials

## Abstract

**Background:**

Extracellular Vesicles (EVs) are sub-micrometer lipid-bound particles released by most cell types. They are considered a promising source of cancer biomarkers for liquid biopsy and personalized medicine due to their specific molecular cargo, which provides biochemical information on the state of parent cells. Despite this potential, EVs translation process in the diagnostic practice is still at its birth, and the development of novel medical devices for their detection and characterization is highly required.

**Results:**

In this study, we demonstrate mid-infrared plasmonic nanoantenna arrays designed to detect, in the liquid and dry phase, the specific vibrational absorption signal of EVs simultaneously with the unspecific refractive index sensing signal. For this purpose, EVs are immobilized on the gold nanoantenna surface by immunocapture, allowing us to select specific EV sub-populations and get rid of contaminants. A wet sample-handling technique relying on hydrophobicity contrast enables effortless reflectance measurements with a Fourier-transform infrared (FTIR) spectro-microscope in the wavelength range between 10 and 3 µm. In a proof-of-principle experiment carried out on EVs released from human colorectal adenocarcinoma (CRC) cells, the protein absorption bands (amide-I and amide-II between 5.9 and 6.4 µm) increase sharply within minutes when the EV solution is introduced in the fluidic chamber, indicating sensitivity to the EV proteins. A refractive index sensing curve is simultaneously provided by our sensor in the form of the redshift of a sharp spectral edge at wavelengths around 5 µm, where no vibrational absorption of organic molecules takes place: this permits to extract of the dynamics of EV capture by antibodies from the overall molecular layer deposition dynamics, which is typically measured by commercial surface plasmon resonance sensors. Additionally, the described metasurface is exploited to compare the spectral response of EVs derived from cancer cells with increasing invasiveness and metastatic potential, suggesting that the average secondary structure content in EVs can be correlated with cell malignancy.

**Conclusions:**

Thanks to the high protein sensitivity and the possibility to work with small sample volumes—two key features for ultrasensitive detection of extracellular vesicles- our lab-on-chip can positively impact the development of novel laboratory medicine methods for the molecular characterization of EVs.

**Graphical Abstract:**

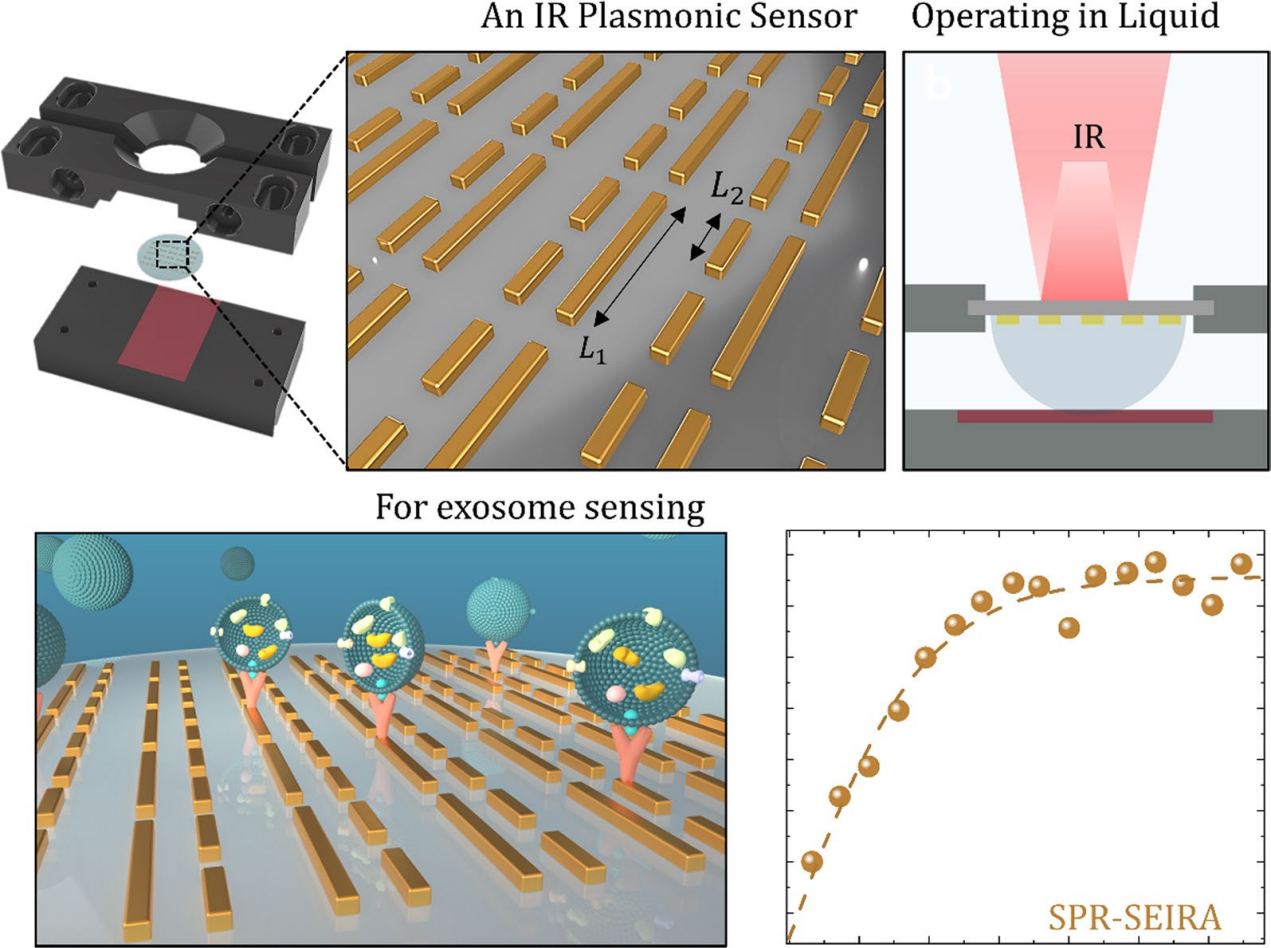

**Supplementary Information:**

The online version contains supplementary material available at 10.1186/s12951-022-01693-2.

## Background

Extracellular Vesicles (EVs) are spherical lipid vesicles with a diameter of tens to hundreds of nanometers released by living cells both in physiological and pathological conditions. They are known to carry in their interior a molecular “cargo” that reflects the conditions of the cell of origin. Because of both their availability in biofluids and the information brought by their molecular cargo, EVs are playing a key role in the development of novel liquid biopsy approaches for cancer diagnosis and therapy monitoring [1–7]. The clinical potential of EVs is further confirmed by the growing number of publications concerning their diagnostic use, which led to the discovery of potential biomarkers for different cancer types [8–12]. Despite this potential, EVs have still not been widely applied in diagnostics, and a change in the paradigm of their analysis is highly demanded to boost their clinical translational process [13].

In this context, Fourier Transform Infrared (FTIR) spectroscopy in the mid-IR wavelength range of 2 to 20 μm is rapidly emerging as a promising tool for molecular profiling of EVs, as it provides label-free biochemical information in terms of their lipid, protein, and nucleic acid content [13–24]. FTIR spectroscopy is especially useful for EV analysis when operated in the attenuated total reflectance (ATR) mode, in which the sensing volume is restricted to an evanescent-wave domain with a thickness of a few hundred nanometers, located at the interface between a total-reflection crystal and either an aqueous solution or its dry deposit [25]. However, the typical transverse focal spot size in ATR-FTIR is of the order of millimeters, and this translates into the requirement that a high number of extracellular vesicles should be present in the starting solution. Another problem of ATR-FTIR is that it measures the chemical content of the entire solution or of its dry deposit, which in the case of serum can include fragments of cell membranes, plasma protein aggregates (e.g., albumin), etc., without any specific sensitivity to EVs and their cargo. In particular, the amide-I IR absorption band, which carries most of the information related to the protein folding state, has to be measured on an almost pure protein population because signatures of all proteins overlap spectrally with each other. Compared to ATR-FTIR, a further reduction of the sensing volume and an increase in specificity towards EV particles would therefore be highly required, for example, by introducing immunocapture techniques in FTIR spectroscopy, as we do here.

Antenna-enhanced IR spectroscopy, performed with lithographic gold nanoantennas fabricated on IR transparent substrates, has been demonstrated in various forms, including the surface-enhanced infrared absorption (SEIRA) effect [26–31]. The formation of intense plasmonic hotspots on the nanoantenna surface and at the nanoantenna resonance frequency in the IR enables a very strong reduction of the required sample volume for sensing. If compared to the surface-enhanced Raman spectroscopy (SERS) [32], SEIRA does not require a distance between the gold surface and the target molecule of the order of the molecule size, and therefore it is more suitable for sensing the molecules in an exosome cargo. A more mature plasmonic technology that is already employed in medical diagnostics is represented by surface plasmon resonance (SPR) sensors working in the visible range, which provide high sensitivity to changes in the refractive index at the metal/liquid interface, however without being able to assess the chemical content of the molecular film [33]. To increase specificity, antibody-antigen pairs are commonly employed in SPR sensing: an antibody monolayer is prepared on the metal surface before exposure to the target solution, in which an antigen is present.

In this work, we provide the proof of concept of a novel plasmonic biosensor for the quantification and molecular characterization of EVs, capable of working both in liquid and dry phases. Similarly to other SPR sensors targeting EVs [13, 34–38], our device relies on immunocapture, allowing us to reduce the detrimental effect of contaminants and select specific EV subpopulations. At variance with conventional SPR biosensors, which mostly function in the UV–VIS range, our device is designed to operate in the mid-IR range of the electromagnetic spectrum, which contains the specific absorption signatures of biomolecules within EVs. For this purpose, we realized and tested a nanostructured metasurface consisting of a periodic array of NanoAntennas (NAs) that combines, in the same lab-on-chip platform, the enhanced chemical specificity of SEIRA and the high quantitative sensitivity of SPR with antibody/antigen pairs. On the one hand, this approach can contribute to achieving a significant improvement in the current EV detection capabilities of FTIR spectroscopy; on the other hand, it permits overcoming the intrinsic lack of chemical specificity in conventional UV–VIS plasmonic sensors.

Interestingly, the combination of SPR sensing and SEIRA nanoantennas has been achieved without any increase in the measurement time duration by exploiting the “multiplex” advantage of FTIR spectroscopy over dispersive spectroscopy: all wavelengths are measured simultaneously in the same interferometric spectral acquisition, taking a few seconds. We have therefore designed plasmonic antenna structures with two main features (i) a dipole resonance frequency in the 1550–1750 cm^−1^ range (wavelengths around 6 μm), which we use for SEIRA spectroscopy of the amide-I and amide-II bands of proteins and (ii) a sharp reflectivity drop at 1800–2200 cm^−1^ i.e., in the transparency window of non-aromatic organic molecules, which we use for SPR sensing to study the reaction dynamics and provide quantitative information on the EV concentration, which is highly relevant in diagnostics. Sample delivery has been engineered through novel fluidic schemes. The device has been tested in a real-world scenario by exposure to EV solutions produced by human cancer cell cultures, which have been thoroughly characterized by different microscopy techniques before the IR sensing experiments. Additionally, we exploited our resonant metasurface to compare the spectral response of EVs extracted from cancer cells with increasing invasiveness and metastatic potential. Our preliminary results show a potential alteration in the average secondary structure content of EVs, estimated by Amide I spectral band deconvolution, whit an increase in β-structures with increasing cell invasiveness and malignancy.

## Results

### Optical characterization of the plasmonic device in air and liquid environment

**Fig. 1:**
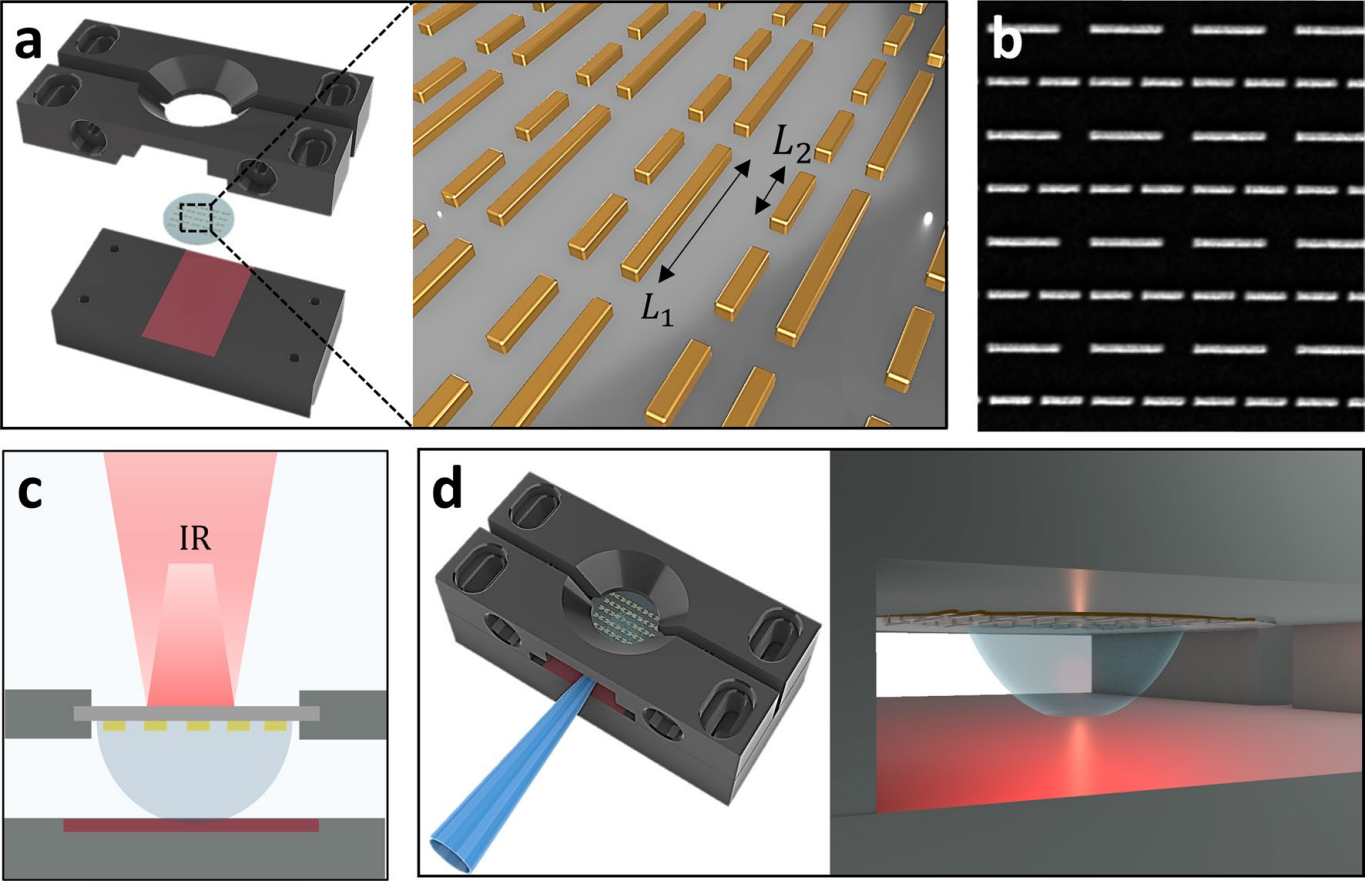
**a** Schematic view of the fluidic-plasmonic device. **b** SEM micrograph of representative double resonant nanoantenna array. **c** Schematic view of the optical setup. **d** Schematic view of the deposition of the 10 μl droplet solution into the fluidic device

In Fig. 1a, we show a schematic view of our integrated fluidic-plasmonic biosensor. The plasmonic part of the device consists of a double resonant dipole nanoantenna array designed to exhibit resonances at two different wavelengths, approximately at 3.5 and 6 μm. Nanoantennas are realized in gold on a transparent CaF_2_ optical window and arranged in 200 μm × 200 μm arrays (see “Materials and methods” section). A Scanning Electron Microscopy (SEM) micrograph of a representative antenna array is reported in Fig. 1b. The optical window is placed in a 3D-printed device holder (Fig. 1a) designed for two main purposes: (i) enabling measurement on a few-microliters droplet, (ii) facilitating the acquisition of real-time reflectance spectra under an FTIR spectro-microscope. Similar to the work of Adato and Altug [26], the plasmonic chip is illuminated from the dry backside of the CaF_2_ substrate while the nanoantennas are dipped in the target solution (Fig. 1c). At variance with the above-mentioned paper, our device holder exploits a wet sample handling technique based on the surface energy contrast between the hydrophobic Teflon-decorated bottom surface of the device (Fig. 1a, red area) and the hydrophilic CaF_2_ window. On the one hand, such hydrophobicity contrast prevents the sample solution from wetting the underlying *Polylactic Acid* (PLA) surface, thus reducing the volume needed to contact the CaF_2_ window, as well as possible sample contamination due to the interaction with the surface; on the other hand, it permits stable pinning of the solution droplet on the sensing part of the plasmonic device, thus allowing for an effortless alignment of the IR beam. To further facilitate the sample droplet deposition, we introduced a slanted surface in the lateral wall of the PLA holder, which is instrumental for guiding the tip of a conventional 20 μL pipette so that the sample droplet is released precisely under the sensing part of the device (Fig. 1d). Droplets can be removed using the same tip guidance structure and substituted without damaging the overlaying nanostructures for the sequential acquisition of background and sample spectra. This experimental setting allowed us to perform experiments using 10 μL droplets, thus substantially reducing the required sample volume compared to more complex and expensive fluidic arrangements.

**Fig. 2:**
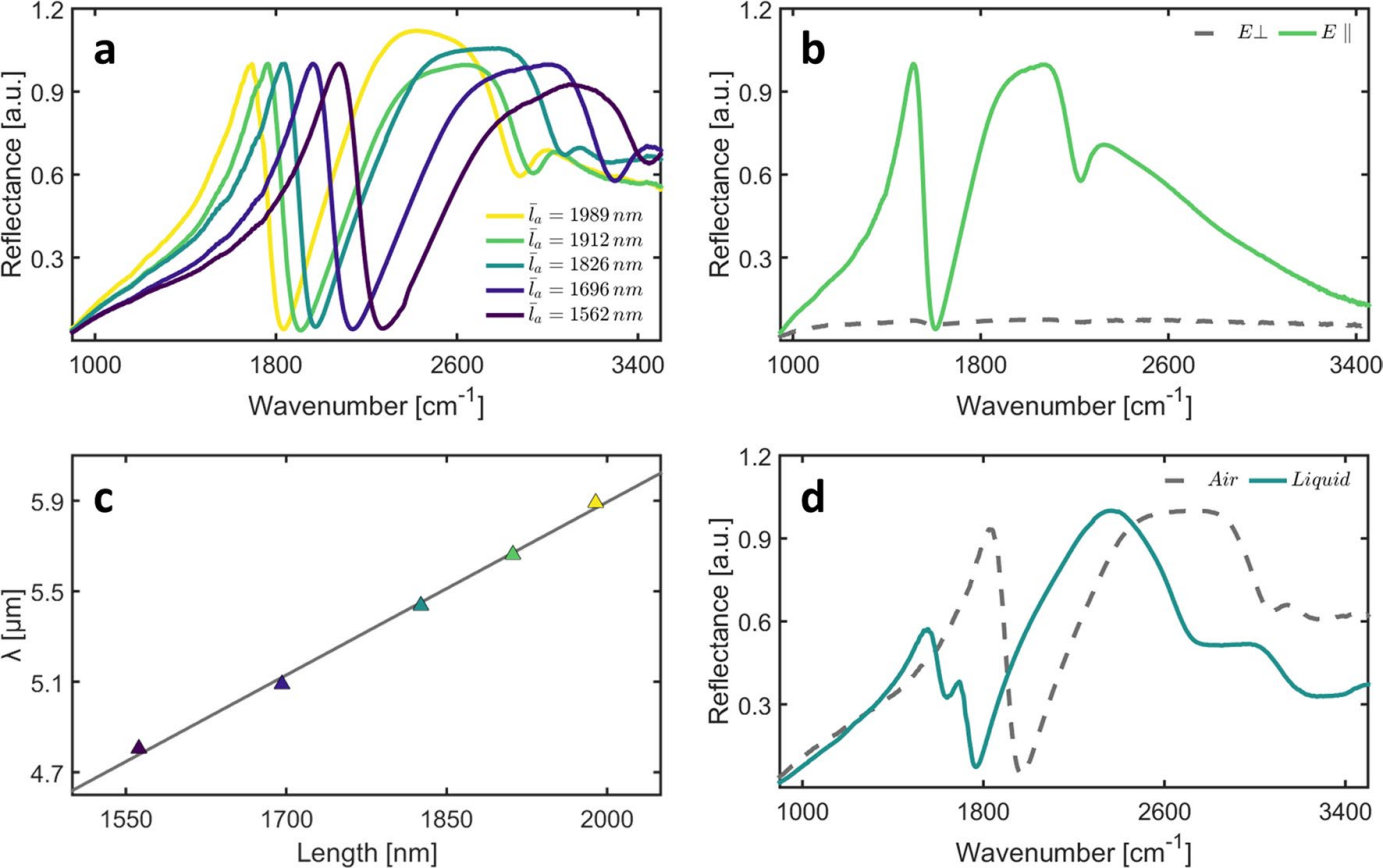
**a** Reflectance spectra of the metasurface as a function of the frequency for different values of the lattice constant. **b** Effect of the incident field polarization. **c** Resonance wavelength as a function of L_1_. **d** Reflectance spectra of a representative nanoantenna array in air and liquid environment

Figure 2a shows a set of reflectance spectra acquired on the fabricated arrays as a function of the nanoantenna length (see “Materials and methods” section). Measurements were performed in air with a blackbody source and referenced to a flat gold area at a different location on the chip surface. The effect of the incident electric field (E) direction was investigated by selectively recording spectra for the two linear polarizations, namely parallel (E_//_) and perpendicular (${\mathrm{E}}_{\perp }$) to the long axis of the rod-shaped antennas. Under E_//_ illumination, two intense antenna resonances can be observed for all the devices, showing substantial overlap with the vibrational signatures of the amide I–II region and the CH stretching bands (Fig. 2a). A comparison between E_//_ (continuous green line) and ${\mathrm{E}}_{\perp }$ (grey dashed line) is reported in Fig. 2b. As expected, for the perpendicular polarization, plasmon resonances are not excited over the investigated spectral range. In Fig. 2c, we investigate the relationship between the resonance wavelength in the amide I-II region and the nanoantenna length, L_1_. Data follow the expected linear scaling in the investigated spectral range 1700–2100 cm^−1^, suggesting that the two sets of rod-like nanoantennas are weakly coupled, and their optical response can be tuned independently by changing L_1_ and L_2_, respectively, as expected [26–28, 39]. The fitted regression line is superimposed on the data. The following fitted slope (m) and intercept (q) were retrieved:$\mathrm{q}=0.62\pm 0.18 \left(\mathrm{p}=0.014\right),\mathrm{ m}=(2.7\cdot {10}^{-3}\pm 1\cdot {10}^{-4}) (\mathrm{p}=2\cdot {10}^{-3})$, both statistically significant. A similar scaling was already demonstrated in previous papers investigating gold nanorods on silicon [27, 31, 39] and CaF_2_ [26, 28, 39, 40] substrate. The above-described linear behavior has a key role in developing SEIRA-based biosensing applications as it offers an effective method to obtain a fine-tuning of the resonance to the vibrational range of interest, in this case, the protein amide-I band at 1660 cm^−1^ and the protein amide-II band at 1537 cm^−1^.

It is worth explicitly pointing out the difference between the devices designed in refs [26] and [28] and our device, despite the similarity of the lithographic design. In these previous works, the electromagnetic design aimed to obtain a double resonance, i.e. two independent dipole antennas of different lengths for sensing two IR bands simultaneously (amide-I of proteins and C–H stretching of lipids). In the present work, we aimed at sensing mainly the amide-I band with the long antenna, and we exploited the Fano resonance between the tail of the short-antenna resonance and the long antenna peak to obtain a sharp spectral reflectivity edge. Sharp spectral edges can be generally exploited for refractive-index sensing of non-absorbing molecules, and indeed the edges in Fig. 2a fall in the 1800–2200 cm^−1^ range in which no absorption of biological molecules is expected. The price paid in our design to enhance the Fano resonance and obtain a sharp edge is that the short antenna resonance is extremely broad, and therefore, the field enhancement in the C-H stretching region (2800–3100 cm^−1^) is weak. To sum up, in our plasmonic design, we obtain both a strong field enhancement in the amide-I range (1570–1720 cm^−1^) and a sharp edge for mass-sensing in the nearby transparency range of biomolecules (1800–2200 cm^−1^), which can then be probed with the same FTIR apparatus. The relatively narrow wavelength window of operation (4.5–6.5 μm) still leaves room for the future substitution of the FTIR spectro-microscope with a more compact tunable quantum cascade laser module [41].

In Fig. 2d, we show the comparison between the reflectance spectra of a selected array measured in air (dashed line) and Dulbecco’s Phosphate Buffer Saline (DPBS) solution (continuous cyan line). The two spectra show similar features, with some notable exceptions. Specifically, the spectrum measured in a liquid environment shows the expected shift associated with the change in the index of refraction of the dielectric half-space, as well as the unavoidable superposition of the broad IR absorption bands of liquid water (centered at 1650 and 3300 cm^−1^). Despite the presence of such absorption bands, the measured reflectance confirms the presence of a significant multiresonant near-field enhancement in the spectral region of interest, thus being suitable for biosensing applications. The described behavior was tested on many devices showing an excellent reproducibility of the optical response both in air and liquid environment.

### Real-time in-situ monitoring of the formation of molecular monolayers

**Fig. 3:**
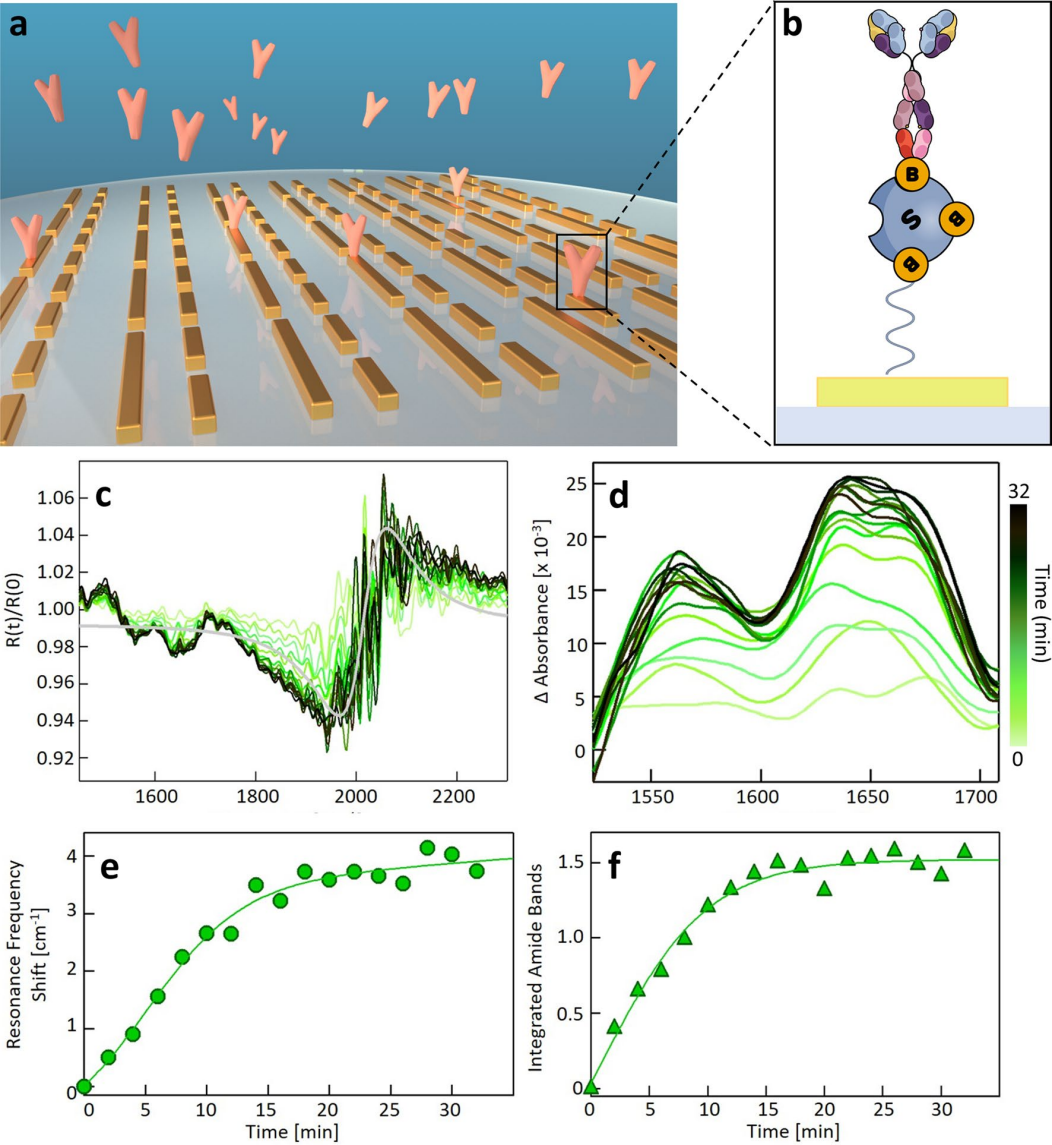
**a** 3D sketch of the nanoantenna array functionalization for EV immunocapture. **b** Schematic representation of the gold-conjugated molecules. **c** The ratio between reflectance spectra R(t), taken at time t after antibody exposure at t = 0, and R(0). The colors, from light green to black represent the increasing t from 0 to 32 min with 2-min steps, as shown in the colorbar on the right. The grey line is a representative S-shaped fitting curve used to evaluate the SPR frequency shift. **d** Detailed view of the different absorption signals in the region of the amide bands (1500–1700 cm^−1^), with the same color legend of the panel c. **e** SPR frequency shift vs. time obtained by the fitting procedure of the data in panel c in the 1800–2100 cm^−1^ range. The solid green line represents the sigmoidal fitting curve. **f** Integral of the difference in absorption signal in the amide band region vs. t and the related sigmoidal fitting curve

The extreme sensitivity of our device and its ability to operate in real-time in a liquid environment is demonstrated by monitoring one of the preliminary functionalization steps required for EV immunocapture (Fig. 3a–f), namely the conjugation of the metasurface with biotinylated anti-CD63, a specific monoclonal antibody with a high affinity for the corresponding tetraspanin (Fig. 3a). As detailed in Fig. 3b, the Au nanorods were first coated with a mixture of biotinylated polyethylene glycol (PEG) molecules conjugated with neutravidin, a tetrameric biotin-binding protein (see “Materials and methods” section). A 10 μL droplet of antibody solution in DPBS was then inserted into the device holder. For this purpose, a 0.025 mg/mL (0.17 μM) solution of biotinylated Anti-CD63 was reconstituted in DPBS. The antibody solution concentration was determined spectrophotometrically in the UV–VIS range by analyzing the protein peak at 280 nm. FTIR reflectance R(t) spectra were acquired in the time interval of 32 min after droplet deposition, with 2-min steps. The reflectance change was computed as a ratio using the spectrum taken at time zero R(0) as the denominator and all other spectra R(t) as the numerator, thus getting rid of the spectral contribution of the previously conjugated biotin and neutravidin molecules. The R(t)/R(0) ratios in Fig. 3c display an s-shaped feature at 1800–2100 cm^−1^, which is related to the refractive index increase after antibody deposition, and two dips at 1560 and 1650 cm^−1^, corresponding to the amide-II and amide-I band center, respectively. The intensity of both the S-shaped feature and the amide dips increases with time after antibody deposition and finally saturates at a plateau level. This clear time-dependent trend can be associated with the progressive functionalization of the nanoantennas with Anti-CD63 molecules, occurring through protein–protein binding interaction (Fig. 3a, b). In Fig. 3d, we show enlarged detail of the absorption difference in the amide I and II region calculated at different times as $\Delta \mathrm{A}=-\mathrm{ln}(\mathrm{R}(\mathrm{t})/\mathrm{R}(0))$. The characteristic protein–protein binding kinetics can be effectively measured by computing the spectral integral of the amide I and II bands after linear baseline correction. The integrated absorbance change (Fig. 3f) shows an abrupt increase in the first few minutes, followed by a slower increase, and it reaches a plateau after t = 10 min. A sigmoidal function is fitted to the experimental points, and the best regression curve is reported. We notice that the time trend displayed in Fig. 3 is qualitatively consistent with the typical trends measured during antibody layer formation with conventional refractive index sensing techniques, such as SPR. Despite their fine mass sensitivity, our sensor displays a key advantage over conventional SPR techniques, as they do not show chemical sensitivity to different molecular species. Conversely, spectroscopic data in Figs. 3d and f show that our sensor is endowed with exquisite chemical sensitivity, which permits the recognition of specific molecular classes, as also shown in previous experimental papers [26, 28].

In Fig. 3e we show the SPR sensing signal measured as the redshift of the zero-crossing point of the S-shaped curve fitting to the 1800–2100 cm^−1^ feature observed in the ratios R(t)/R(0) of Fig. 3c. Interestingly enough, this temporal trend appears to be highly correlated to the one displayed in Fig. 3f, with an abrupt increase occurring in the first 10 min, followed by signal saturation. This highly correlated behavior between SPR sensing and Amide bands chemical sensing is not surprising for a pure protein sample like the one used for the described functionalization. Additionally, the data in Fig. 3 have allowed us to estimate the molar sensitivity of our plasmonic device. In our case, absorption arises from a 100 µm × 100 µm area in the focal plane, which is defined by the microscope's numerical aperture and the size of the array. The physical gold structure of the long nanoantennas (L_1_ in Fig. 1a) covers about 10% of this region, which gives us an area of approximately 10^–9^ m^2^. This interaction region should be further reduced considering that the field enhancement is not homogeneous over the antennas’ length, leading to a smaller filling fraction of the plasmonic hotspots. For this reason, the device sensitivity was estimated by considering only the detection volume at the close vicinity of the nanorod tips. The extension of this region has been estimated through electrodynamic simulation of our nanostructures with the Green dyadic method (GDM) performed for an incident field of 6.050 µm [42], which showed the occurrence of a significant field enhancement over a 150 nm range from the tip end, resulting in two approximately semi-spherical plasmonic hotspots (Additional file 1: Figs. S1 and S2), in agreement with [26]. Considering the antenna length L_1_ of 1800 nm (Fig. 1g), we obtain an effective sensing volume of approximately 1/6 of the solution volume above the whole nanorod. Considering an evanescent field extension of approximately 20 nm above the nanoantenna surface approximately matching the antibody monolayer thickness, we obtain an effective interaction volume of the order of 0.3∙10^–13 ^L. In this volume, we can estimate the presence of approximately $5.1\pm 0.5$ zeptomoles of protein ($\mathrm{n}=1.7\cdot {10}^{-7}\mathrm{M}\cdot {0.3\cdot 10}^{-13}\mathrm{ l}=5.1\cdot {10}^{-21}$ moles). This estimation places the realized device among state-of-the-art SEIRA sensors available in the literature [27].

### Real-time cancer-derived EV detection in a liquid environment

In this paragraph, we investigated the possibility of using our plasmonic-fluidic platform to detect EVs in real time in a liquid environment. For this purpose, we purified exosomes from HT29 human cancer cells [43], which were chosen as a model system. This cell line is widely used to study different aspects of the biology of human cancer, with emphasis on colorectal cancer, but has also attracted a lot of attention thanks to their capability of expressing characteristics of mature intestinal cells, such as enterocytes or mucus-producing cells, including the gene expression of transporters and metabolic enzymes [43–48]. The biomedical relevance of the HT29 cell line for the study of extracellular vesicles is further stressed by strong experimental evidence demonstrating that HT29-derived EVs play a key role in enhancing the liver metastatic potential of Caco2 colorectal cancer cells in murine models of colorectal liver metastasis [49].

**Fig.4:**
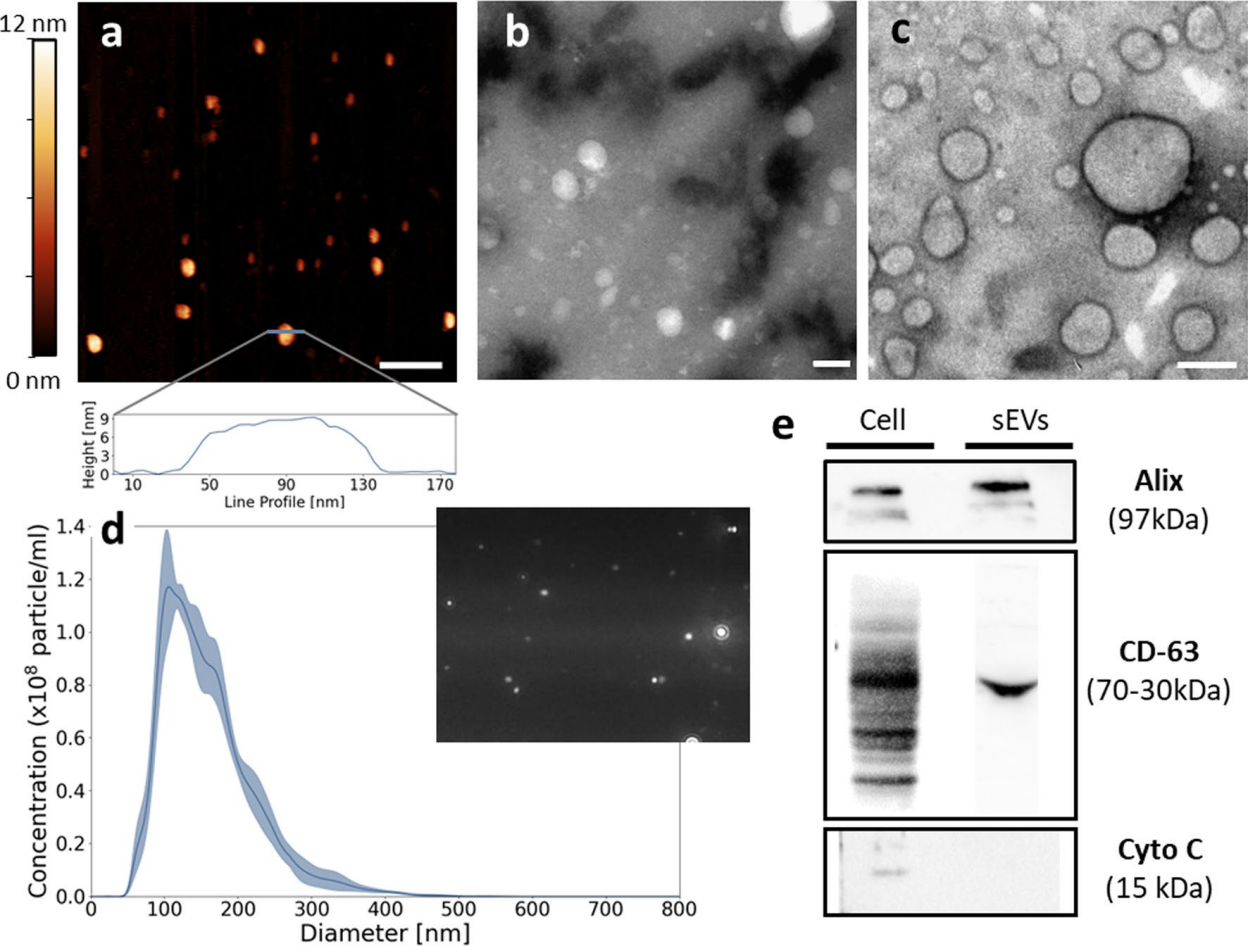
Characterization of the EVs isolated from HT-29 cells. **a** Representative AFM topography of purified EVs together with a selected line profile. (scale bar 300 nm). **b**, **c** Representative TEM images of purified EVs. Scale bars 80 nm (**b**), and 50 nm (**a**). **d** Nanoparticle tracking analysis of EVs isolated from HT-29, size distribution with a video frame. **E** Western blot analysis of EVs markers (Alix, CD63) and non-EVs marker (Cytochrome C) in HT29 cell-derived EVs and whole-cell lysates

We cultured the HT29 cancer cells in exosome-depleted fetal bovine serum (FBS) to prevent the presence of EV contaminants derived from serum, carrying out three identical replicates of the experiments, leading to consistent results. A representative SEM micrograph of the cultured cells is reported in Additional file 1: Fig. S3. The precipitated EVs were characterized with Atomic Force Microscopy (AFM, Fig. 4a), Transmission Electron Microscopy (TEM, Fig. 4b, c), Nanoparticle Tracking Analysis (NTA, Fig. 4d), and Western blot (Fig. 4e). Figure 4a shows a representative AFM topography of the extracted nanoparticles (top), together with a selected height profile (down). A characteristic "cup-shape" morphology, with an aspect ratio of 8–10 can be observed, as reported in the literature in similar conditions [50]. Two representative TEM images with different magnifications (Fig. 4b, c) acquired on the same sample type show the expected EV morphology, which consists of round-shaped nanoparticles surrounded by double-layer membranes. The NTA allowed an evaluation of the EV diameter distribution (Fig. 4d), which shows a modal size of approximately 100 nm, consistent with literature data on EVs extracted from HT29 cells [51]. Finally, Western blot has been used to confirm the presence of extracellular vesicles. Figure 4e shows EV markers (CD63 and Alix) in the nanoparticles composing the pellet obtained from HT-29 through the precipitation process. Conversely, the negative marker cytochrome c is detectable only in the whole cell lysates while is absent in the EV samples, as expected.

**Fig.5:**
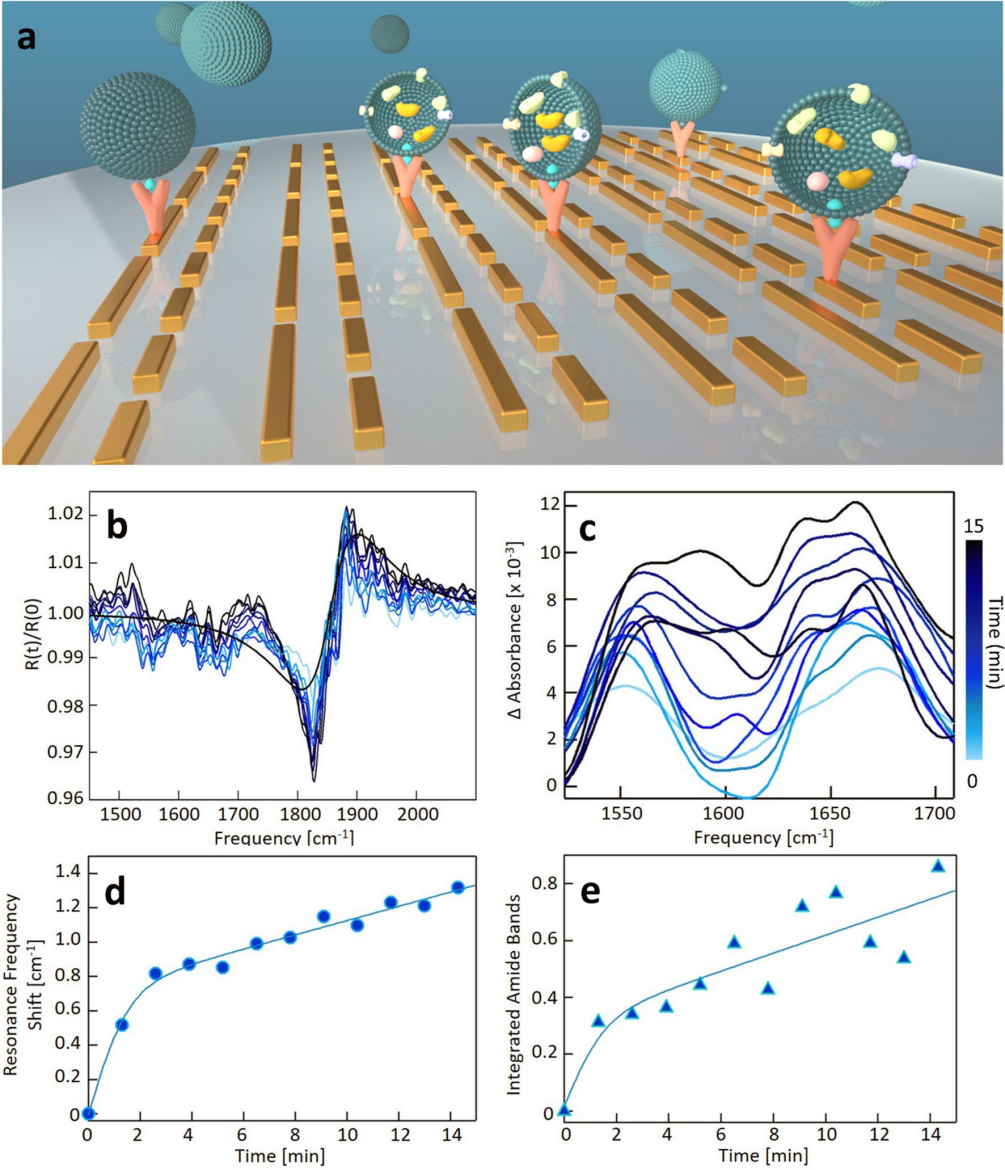
**a** 3D sketch of the small extracellular vesicles immunocapture on the functionalized nanoantenna array. **b** Ratio between reflectance spectra R(t), taken at time t after EVs exposure at t = 0, and R(0). The increasing blue color intensity represents the increasing t from 0 to 14 min with 1.3-min steps. The black line is a representative S-shaped fitting curve used to evaluate the SPR frequency shift. **c** Detailed view of the difference absorption signal in the region of the amide bands (1500–1700 cm^−1^), same color legend of panel b. **d** SPR frequency shift vs time obtained by the fitting procedure of the data in panel b in the 1700–2000 cm^−1^ range. The solid blue line represents the sigmoidal fitting curve. **e** Integral of the difference absorption signal in the amide band region vs t and the related sigmoidal fitting curve

For the IR-sensing experiment, a 10 μL solution droplet derived from samples characterized in Fig. 4 was inserted into the fluidic holder containing our Anti-CD63-conjugated nanostructures. The time-dependent EV immunocapture, schematically represented in Fig. 5a, is monitored through the same spectral quantities defined in Fig. 3, namely: the $\mathrm{R}(\mathrm{t})/\mathrm{R}(0)$ ratio (Fig. 5b), the baseline-subtracted absorption difference in the amide I–II bands $\Delta \mathrm{A}(\mathrm{t})=-\mathrm{ln}(\mathrm{R}(\mathrm{t})/\mathrm{R}(0))$ (Fig. 5c), and the two sensorgrams obtained from the SPR shift (Fig. 5d) and the integrated $\Delta \mathrm{A}$ intensity (Fig. 5e).

As expected and similar to Fig. 3c, $\mathrm{R}(\mathrm{t})/\mathrm{R}(0)$ signals are s-shaped about 1800—2100 cm^−1^ and display two dips in the 1500–1700 cm^−1^ spectral range, which are related to the RI increase and the amide I–II bands of EV proteins, respectively (Fig. 5b). The zero-crossing point in the 1800–2100 cm^−1^ range of Fig. 5d shows an abrupt increase in the first 2 min, followed by a slower, and approximately linear, increase afterward, which can be modeled with the sum of a line and a sigmoidal function (continuous black curve in Fig. 5d). As discussed in the Additional file section (Additional file 1: Fig. S4), where we provide additional experimental evidence, the first abrupt increase in the NA redshift is likely to be correlated to the specific interaction between NAs and EVs mediated by CD63-immunocapture, while the subsequent linear increase is probably related to unspecific interaction with the unconjugated substrate surface. Very interestingly, the integrated $\Delta \mathrm{A}$ intensity (Fig. 5e) displays a similar temporal trend, with an abrupt increase in the first 2 min, followed by a roughly linear increase. Despite the similarities between these time trends, a lower noise can be observed in the SPR shift (Fig. 5d) compared to the integrated $\Delta \mathrm{A}$ (Fig. 5e). Such noise difference has not been observed during the device functionalization with antibodies (Fig. 3e vs. Fig. 3f). This finding can be explained in terms of the different biochemical compositions of antibodies that are 100% proteins, and exosomes, which contain different molecular classes (proteins, lipids, carbohydrates, and nucleic acids) and therefore can show a varying protein content. In the Additional file 1 section, we provide a preliminary estimation of the minimum number of measurable EVs with our devices, which is of the order of several hundreds of particles. This estimation is obtained by using experimental findings in the literature [52], the electrodynamical simulation in Additional file 1: Fig. S2, and the estimated protein sensitivity in the previous paragraph.

### Plasmonic-aided IR characterization of EVs derived from cancer cells with increasing invasiveness and metastatic potential.

This paragraph aims at evaluating the applicability of our plasmonic metasurface to the study of EVs derived from cancer cells with different degrees of invasiveness. In this context, the investigation of the same cancer cell line differentiated in an epithelial or mesenchymal phenotype is extremely relevant, as the epithelial-to-mesenchymal transition (EMT) plays a key role in tumor progression and metastatic expansion [53–56]. As a model system, we used human colorectal adenocarcinoma Caco-2 cells, which represent an effective model of differentiation as they can be gradually shifted upon chemical treatment from a proliferative and aggressive mesenchymal phenotype to a less invasive epithelial one. For this purpose, we exploited an n-acetyl-l-Cysteine (NAC) based treatment exerting an inhibitory effect on the activation of c-Src tyrosine kinase, a widely studied proto-oncogene, whose expression and activity is increased in several malignant conditions [57]. Among the wide range of c-Src functions, particularly relevant for our model is its ability to disassemble cell–cell junctions by phosphorylating E-cadherin and β-catenin, and promoting the translocation of the latter in the nucleus, where it exerts an oncogenic action [55, 58, 59]. The effectiveness of this treatment was already demonstrated in-depth in previous papers from some members of our group, verifying the occurrence of multiple epithelial markers upon NAC treatment. These markers include the formation of cell–cell junctions accompanied by an increase in the expression of E-Cadherin and a β-catenin relocation, the arising of a regular polygonal cell morphology showing the typical brush-border microvilli and a general switch in gene expression from mesenchymal to epithelial phenotype [55, 58, 60].

**Fig. 6:**
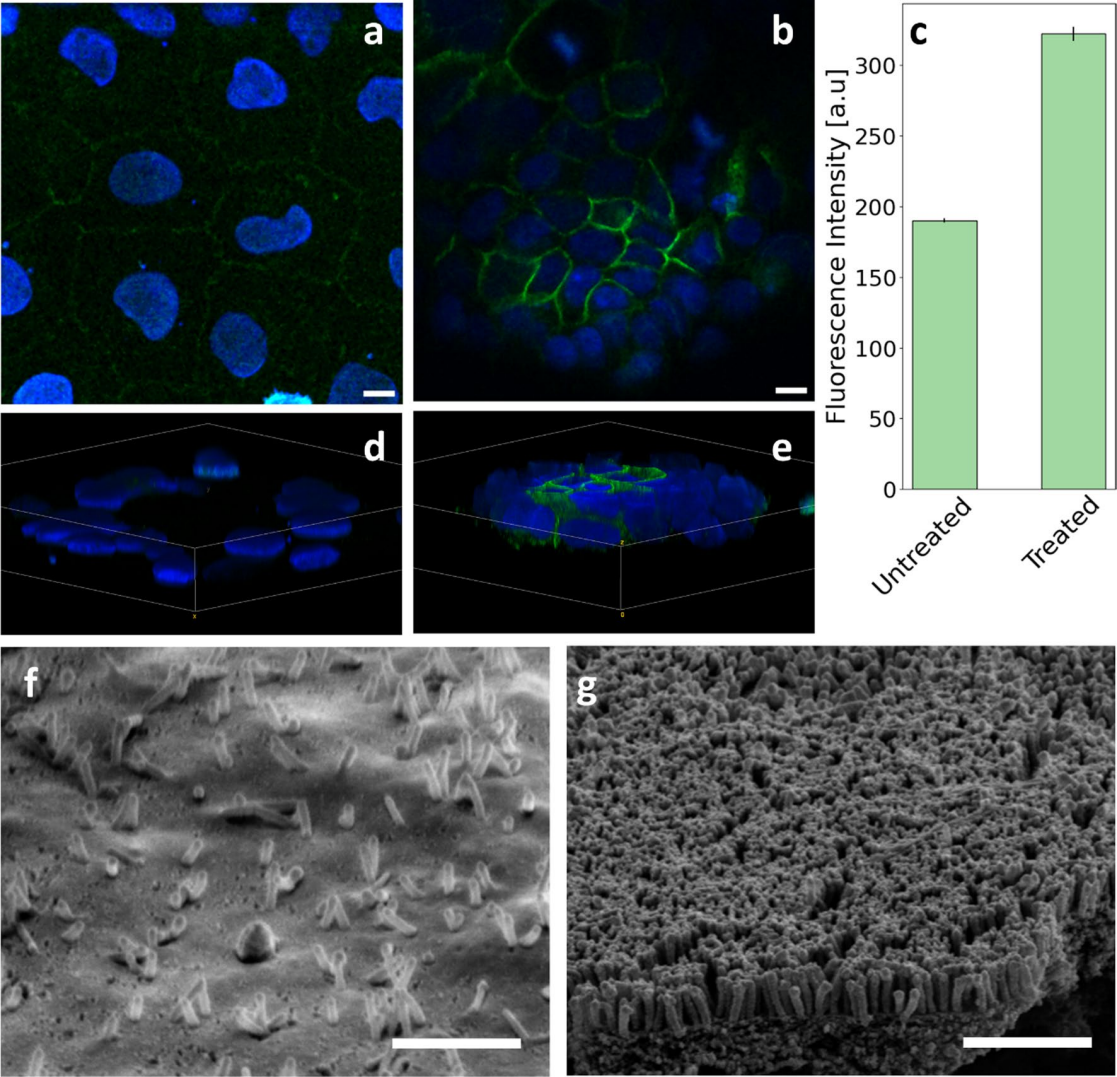
Representative CFM images of untreated (**a**) and NAC-treated (**b**) Caco2 cancer cells. Scale bar 10 µm. **c** comparative analysis of E-cadherin expression in the two sample types obtained by computing the average green fluorescence intensities on cell-to-cell profiles using tens of CFM images. Representative 3D reconstructions from confocal image stacks for untreated (**d**) and treated (**e**) cells, respectively. Representative SEM micrographs of the cell surface for untreated (**f**) and treated (**g**) cells. Scale bar 2 μm

Before EVs extraction and analysis, we confirmed the occurrence of cell differentiation upon NAC treatment (10 mM) using confocal fluorescence microscopy (CFM) and scanning electron microscopy (SEM). In Fig. 6a, b, two representative CFM images of the two cell phenotypes are shown. In these images, E-cadherin was stained with Alexa-488 and cell nuclei with Hoechst (see “Material and Methods” section). Qualitative observation of Fig. 6a, b shows clear changes in cell shape and arrangement, accompanied by a remarkable increase in the E-cadherin expression at the cell–cell junctions upon NAC exposure (6a) compared to the untreated counterpart (6b). The increased E-cadherin expression in the treated sample is quantitatively verified in Fig. 6c, where we report a bar plot analysis obtained averaging the green fluorescence intensity of hundreds of cell-to-cell profiles, from tens of independent CFM images. The method used to segment nuclei for cell recognition and pick the cell-to-cell profiles is widely described in Romanò et al. [54]. Data are reported as mean ± sem. Two representative 3D reconstructions from confocal image stacks are reported in Figs. 6d and e for untreated and treated cells, respectively. In the untreated sample (6d), a layer of flat cells can be observed, consistently with the occurrence of a mesenchymal phenotype. On the contrary, in the NAC-treated sample, cells tend to assume a cuboid-like shape, accompanied by an intense expression of E-cadherin at the cell–cell junctions, consistently with the presence of a more epithelial phenotype. Two representatives SEM micrographs of the cell surface for the untreated and NAC-treated samples are reported in Fig. 6g and f, respectively. Consistently with the occurrence of an epithelial phenotype, the cell surface of the NAC-treated sample displays the de-novo formation of the typical brush-border membrane, composed of microvilli with a thickness of about 100 nm and a length of a few hundred nanometers. Conversely, cells in the untreated sample display a smoother surface, consistently with the occurrence of a more mesenchymal phenotype.

**Fig. 7:**
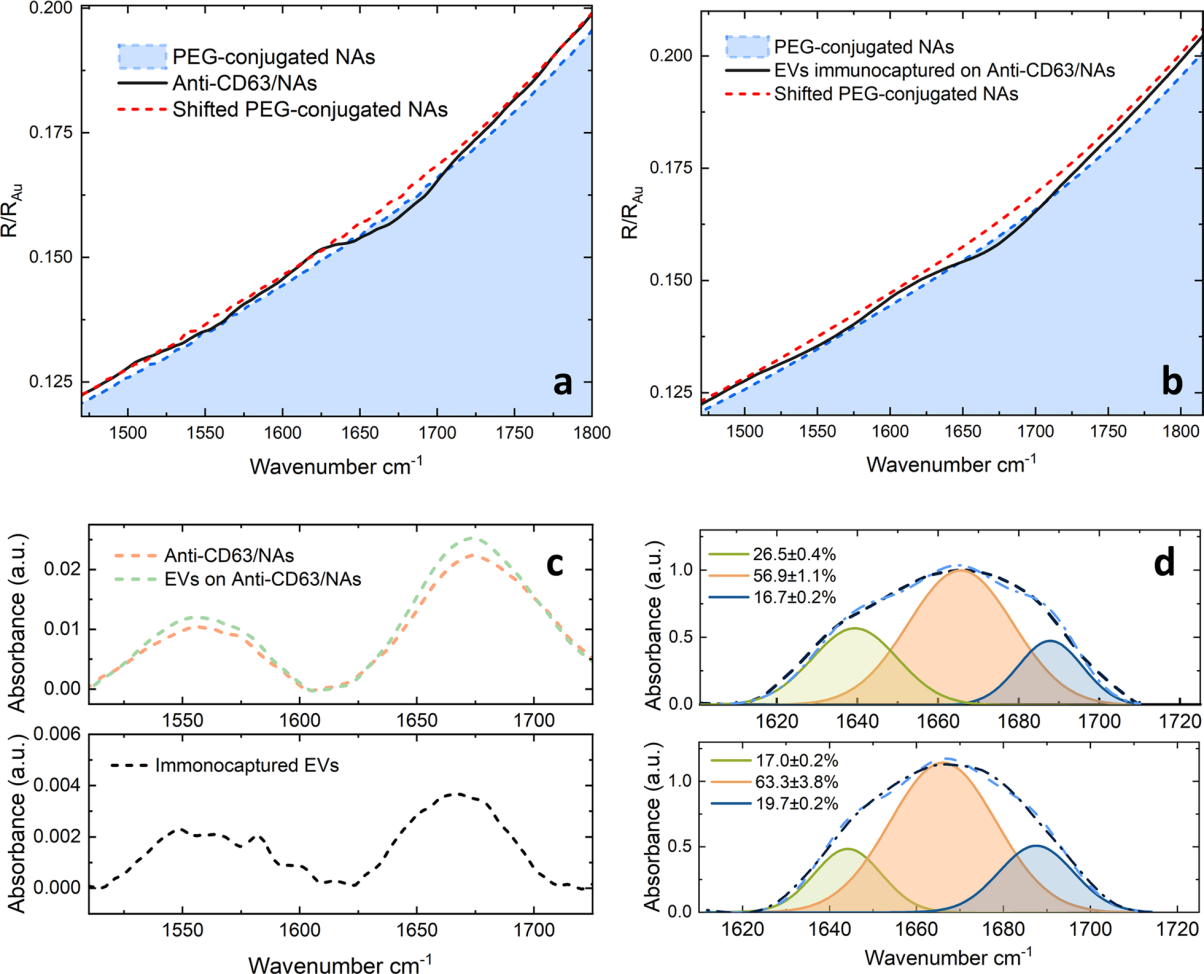
**a** Enlarged detail of the reflectance of our PEG-conjugated metasurface in the 1430–1800 cm^−1^, spectral range measured in a dry environment (blue dashed line), together with the spectral response of the same sample after Neutravidin and Anti-CD63 functionalization (black solid line) and the red-shift compensated reflectance of the PEG conjugated metasurface. **b** enlarged detail of the reflectance of our PEG-conjugated metasurface in the 1430–1800 cm^−1^environment (blue dashed line), together with the spectral response of the same device after EV immunocapture and the red-shift compensated reflectance of the PEG conjugated metasurface. **c** Absorbance in the Amide I/II region of Anti-CD63 and Neutrovidin molecules with (green dashed line) and without (orange dashed line) immunocaptured EVs. **d** Enlarged detail of the Amide I region for EVs extracted from the Caco2 cells in the Epithelial Phenotype (upper panel) and the mesenchymal Phenotype (lower panel). A peak deconvolutional analysis with gaussian fits is superimposed on each plot

In Fig. 7, we investigate the spectral response of EVs extracted from the two cancer cell phenotypes, namely the more aggressive mesenchymal phenotype and the less invasive epithelial one. For the device functionalization, we adopted the same protocol described in Figs. 3 and 5, namely gold NAs were functionalized with a molecular sequence composed of a mixture of PEG molecules, Neutravidin, and Anti-CD63, which were subsequently used for EV immunocapture. Each functionalization step was carried out in a liquid environment, as previously described. After each step, the patterned metasurface was rinsed in de-ionized water, dried under nitrogen flux, and measured in the PIR geometry (Fig. 1c) with an unpolarised IR source. In Fig. 7a, we show enlarged detail of the spectral response in air of our PEG-conjugated gold NAs in the 1480–1800 cm^−1^ spectral range (blue dashed line). Consistently with Fig. 2d, NAs’ reflectance shows the expected spectral behavior and the absence of specific absorption signals of proteins in the Amide I/II region. The device reflectance measured after Anti-CD63 functionalization is reported as a black continuous line in the same figure. As expected, the qualitative observation of the aforementioned curve highlights two features that confirm device functionalization: (i) the presence of the typical absorption signature of Amide I and II bands, (ii) the occurrence of a redshift of the spectral NA response of approximately 10 cm^−1^. The same analysis is carried out after EV immunocapture in Fig. 7b, where we show the reflectance of PEG-conjugated NAs before (blue dashed line) and after EV immunocapture (black continuous line). To obtain data in Fig. 7b, we used EVs extracted from Caco2 cells in the epithelial phenotype. Similarly to the previous case, functionalized NAs show the presence of marked absorption signatures in the Amide I and II band and a redshift of approximately 12 cm^−1^, due to the local changes in the index of refraction. A comparative analysis of Fig. 7a and b show that both, the redshift and the Amides I/II absorption intensity are greater in Fig. 7b compared to Fig. 7a. This was an expected result as, similarly to Fig. 7a, the metasurface in Fig. 7b is conjugated with neutravidin and Anti-CD63. In addition, Fig. 7b shows the contribution of the captured EVs which enhances the plasmonic redshift and deepens the signature of protein absorbance. Starting from these measurements, we computed the Absorbance A of captured molecules by using the equation $A=-ln\left(\frac{R}{{R}_{0}}\right),$ where R is the reflectance of functionalized NAs and R_0_ is the initial reflectance of PEG-conjugated Nanoantennas after redshift compensation (red dashed lines in Fig. 7a, b). The computed Amide I/II signals are shown in the upper panel of Fig. 7c for Neutravidin and Anti-CD63 (orange dashed line) and Neutravidin, Aniti-CD63, and EVs (green dashed line). Concerning Fig. 7c, a *caveat* is necessary, as we are not interested in the cumulative absorbance of Neutravidin, Anti-CD63, and EVs, but rather in the absorption signature arising from a pure EV sample, which is computed in Fig. 7c (lower panel) by subtracting the two mentioned curves. A linear baseline was removed from the spectrum after curves subtraction. Interestingly, Fig. 7c (lower panel) displays the typical shape of amide I and II bands, suggesting the possibility to perform a quantitative analysis of the Amide I band shape to estimate the average secondary structure content of proteins within EVs. In Fig. 7d, we show enlarged detail of the amide I for EVs extracted from Caco2 cancer cells in the mesenchymal (upper panel, black dashed line) and epithelial phenotype (lower panel, black dashed line).

To estimate the average secondary structure contentment within bonded EVs, we used Amide I band deconvolution, a quantitative analysis technique that permits to separate merged absorption signatures through the identification of inverse peaks in the second derivative spectra, followed by Gaussian fitting to quantify the corresponding intensity of the absorption signals contributing to the band shape. This procedure was carried out using the software package Origin Pro 2022, which is endowed with a dedicated application for peak deconvolution. Three contributions were highlighted, which are centered at approximately 1640 cm^−1^ (assigned to native beta-sheets), 1665 cm^−1^ (assigned to α-helix and random coil structures), and 1680 cm^−1^ (assigned to the antiparallel β-sheets and β-turns). These assignments are in close agreement with those used for deconvolving the Amide I band of complex protein aggregates measured with nano-IR [61]. The three corresponding Gaussian curves are reported in green, red, and blue, respectively, and are shown together with the cumulative fitted curves (blue dashed line). The perceptual absorption intensities of the three types of secondary structures were also reported in the plot. Interestingly, we found that EVs derived from the more aggressive and malignant cell phenotype contain a higher percentage of β structure, compared to the epithelial phenotype, as quantified by means of the alpha over beta ratio (α/β) according to [62]. More specifically a value of $\frac{\alpha }{\beta }$ =1.3 ± 0.03 and 1.73 ± 0.10 are obtained for the epithelial and the mesenchymal phenotype, respectively (Z = 3.73, p = 0.00019 for a two-tailed Z test).

## Discussion

It is well known that cancer genesis and development affect protein abundance, structure, conformation, and dynamics. As such, the investigation of cancer-induced protein heterogeneity has a key role in the search for disease biomarkers, in the understanding of pathogenic mechanisms, as well as in drug development [63–66]. Protein heterogeneity in cancer-derived EVs has been strongly correlated with tumor malignancy, progression, and metastatic potential, stimulating the search and validation of novel liquid biopsy approaches for cancer detection, staging, and therapy monitoring. In this context, while large-scale proteomic studies unveiled remarkable alterations in the protein content of tumor-derived EVs [64, 67, 68], the protein conformational state appears to be less investigated. To tackle this issue, we presented here the proof of concept of a fluidic-plasmonic biosensor for EV quantification and molecular characterization, also capable of providing quantitative information on the EV protein conformational state.

The combined use of plasmonic and fluidic biosensors has been already demonstrated in EV research, leading to the realization of effective devices with potential diagnostic applications capable of evaluating the expression of protein markers on the EV surface [13, 34–38]. Most of these devices operate in the UV–VIS range and exploit the SPR effect for EV quantification after immunocapture. At variance with these devices, our lab-on-chip is the first plasmonic EV sensor designed to operate in the mid-IR range of the electromagnetic spectrum. The key feature of our device is the combination of SPR sensing with SEIRA nanoantennas (Figs. 3, 5, and 7). Similarly to UV–VIS sensors, we exploited the SPR effect for EV quantification after immunocapture, a clinically valuable piece of information as tumorigenesis affects many pathways regulating EV release. EVs are indeed shed by tumor cells in higher numbers in comparison to normal cells, and a higher concentration is thus associated with increasing tumor mass or severity, making EV quantification extremely important as a prognostic biomarker [1–7, 36, 69–77].

Our sensor goes beyond simple mass characterization, as it is specifically designed to target the mid-IR Amide I and II absorption bands of proteins, which provide quantitative information on protein conformational state in terms of the average secondary structure content [78–81]. Notably, this information can be obtained in a non-destructive fashion, leaving the EV lipid shell intact, which is especially important for membrane proteins, whose separate characterization would require the use of perturbative treatments with detergents. Although this non-destructive characterization could be in principle obtained with conventional ATR-FTIR spectroscopy [15, 54, 78–85], our device offers several advantages in terms of sensitivity and sample purity. The increased sensitivity of our methods is provided by the strong electromagnetic field enhancement provided by the SEIRA effect. More specifically, the incident electromagnetic field in our device is amplified from several hundred to more than a thousand times close to the resonant nanostructures, as estimated with electro-dynamical simulations based on the Green Dyadic Method [42] (Additional file 1: Figs. S1 and S2). To fully exploit this field enhancement, we take the advantage of immunocapture, which increases EV concentration along the nanorods, reducing the water content in the close vicinity to the surface, where the field enhancement is maximum. At the same time, immunocapture strongly limits the detection of non-EV particles related to purification methods. EV extraction from complex media is, in fact, a challenging task as these molecules are highly heterogeneous in size, content, function, and origin, and the purified samples might be affected by a wide range of contaminants [86–89]. Thanks to the synergistic effect of SEIRA and EV immunocapture, we managed to monitor the dynamical interaction of EVs with the sensing part of the device through the acquisition of the specific spectral fingerprint in the amide I/II region of proteins within extracellular vesicles.

To further stress the potential diagnostic relevance of studying the Amide I/II band shape of EVs with our device, we carried out a comparative analysis of EVs derived from intestinal cancer cells with different degrees of invasiveness and metastatic potential. For this purpose, we used a NAC-based pharmacological treatment on Caco2 intestinal cells, able to precisely control the Epithelial-to-Mesenchymal (EMT) transition in-vitro (Fig. 6), a key process for tumor expansion and metastasis. Thanks to this treatment, we extracted EVs from Caco2 cells with different phenotypes, namely a more invasive mesenchymal phenotype, with higher metastatic potential, and a less motile epithelial one. Thanks to the SEIRA intense field enhancement, we obtained a high-quality signature of the EV amide I bands, suitable for estimating the average secondary structure content within the two EV types in terms of α and β structures. This has been done through band deconvolution into separate Gaussian components. Interestingly enough, we found that EVs derived from the most aggressive phenotype display an increased content in β structures, compared to their epithelial counterpart. Although this has to be considered a preliminary result, which needs to be confirmed on a more statistically robust sample, we believe that it is worth to be commented more in-depth, as similar FTIR results have been already reported measuring blood samples and tissue biopsies, supporting the idea that the average protein conformational state that can be measured with our device is a potential source of novel quantitative EV biomarkers of cancer. For instance, Wang et al. detected a markedly reduced α-helix/β-sheet ratio in serum extracted from lung cancer patients compared to control subjects [90]. Analogous finding was demonstrated on murine models by Yamada et al. [91]. Dong et al. analyzed tissue biopsies of CRC patients and control subjects, showing that malignant tissues have more beta than alpha structures compared to normal tissues [62], consistently with our results on EVs. In the same paper, the authors also demonstrate that more than 60% of FTIR data variability between malignant and benign tissues is due to changes in the Amide I/II region, which is also interesting for our purposes as it confirms the potential clinical utility of our device, which is specifically designed to detect these changes in an ultrasensitive fashion.

Besides sensitivity, our device has further advantages over bulk spectroscopic techniques, which are related to the possibility of selecting clinically valuable EV subpopulations. Before discussing this point, we stress that, for the proof of concept of our lab-on-chip, we used a single EV marker, CD63. However, using the same functionalization strategy, our nanorods could be conjugated with different antibodies for different EV surface markers such as EpCAM, which is overexpressed in many types of cancers [92–97] or claudin 1,7, and Cadherin-17, which are specific for colorectal cancer [92, 96–98]. Targeting these markers would be instrumental for discriminating healthy subjects from cancer patients or for metastasis detection [97]. Additional examples could be the possibility of measuring the expression of PD-L1 on the EV surface to assess patient eligibility for immunotherapy with checkpoint inhibitors or the detection of the EV surface protein GPC1, which is overexpressed in pancreatic cancer [99]. We believe that the possibility of selecting specific EV subpopulations, each with its peculiar response in the Amide I and II bands, could provide scientists and physicians with a further degree of freedom in the search for novel cancer biomarkers.

To conclude, it is worth commenting more in-depth on the relevance of the adopted fluidic scheme, especially for the EV characterization in a liquid environment. Most of the previously published lab-on-chip platforms for EV characterization rely on the use of micro-channels fabricated on polydimethylsiloxane (PDMS), which represents the gold standard in this field [13, 34–38]. Nevertheless, PDMS devices have some limitations, such as a labor-intensive fabrication process [100] and problems due to leakage and/or unbalanced pressure among chambers [101]. Air bubbles removal is a further limitation in closed PDMS devices, which can be overcome by using effective but complex fluidic designs that might include open channels and/or semipermeable membrane for degassing [77, 102–106]. This issue is particularly relevant for IR plasmonics, as local thermally-driven outgassing is a confounding agent in interpreting results. In this context, 3D printing has emerged as an alternative fabrication method, having the potential to streamline microfluidics in a higher number of laboratories [100, 107–109]. Here, we realized a 3D-printed PLA sample holder, which permits stable pinning of a sample solution droplet on the sensing part of the device, allowing us to easily conduct measurements in the Plasmonic Internal Reflection (PIR) geometry. This geometry has been already demonstrated for SEIRA in the pioneering papers of Adato et al. and Rodrigo et al. [26, 28], but—to the best of our knowledge—this is the first time that a single 10 μL droplet is used for this purpose. To use such a small volume, our device exploits the surface energy contrast between its hydrophobic bottom surface and the hydrophilic CaF_2_. Similar to other wet-sample handling tecniques [31, 40, 110–112], our device not only reduces working volumes but also prevents contaminations due to the interaction of the biomolecular solution with the holder walls. Furthermore, it is reusable after cleaning, naturally fosters degassing, and can be quickly realized on-demand in a single fabrication step.

## Conclusion

In this paper, we have developed and tested an SPR-SEIRA lab-on-chip for the detection and characterization of EVs derived from human cancer cells with potential future application in diagnostics. The device is capable of working in the photonic internal reflection mode using a sample solution droplet of 10 μL containing EVs, thus significantly reducing the required sample volume compared to more complex fluidic devices. The plasmonic sensor has a double-resonant design, consisting of two sets of parallel nanoantennas of different lengths. Similar to other SIERA devices, the long nanoantenna in our biosensor is designed to target the Amide I and Amide II bands of the mid-IR wavelength spectrum. At variance with previous SEIRA sensors*,* we exploited the Fano resonance between the tail of the short and the long nanoantennas’ peaks to obtain a sharp spectral reflectivity edge in the 1800–2200 cm^−1^ range, which is used for SPR-based mass sensing. Thanks to this design, the device permits real-time monitoring of the metasurface functionalization with antibodies and the subsequent EV immunocapture. A protein sensitivity down to the zeptomoles range is demonstrated. Most importantly, the sensor allows for measuring the specific IR spectral fingerprint of EVs in the 1500–1700 spectral range, which provides clinically relevant information on the protein content and conformational state. This capability was further tested on EVs derived from cancer cells with a different degree of invasiveness, showing differences in the average secondary structure content of proteins within the EV cargo. Thanks to the high protein sensitivity and the possibility to work with small sample volumes—two key features for ultrasensitive detection of extracellular vesicles- our lab-on-chip can positively impact the development of novel laboratory medicine methods for the molecular characterization of EVs.

## Material and methods

### Lab-on-chip fabrication and functionalization

The Mid-IR nanoantenna sensor was realized by Electron Beam Lithography (EBL) direct writing on 1-mm thick, optical grade CaF_2_ substrate (by Crysel). The antenna array with nm-size precision was patterned on a 300 nm thick Poly(methyl methacrylate) (PMMA) EBL resist layer. To prevent resist and substrate charging during the e-beam exposure, a phenomenon leading to deformations of the nanoscale antenna definition, the PMMA resist layer was coated with a thin (5 nm) Cr layer. Such charge compensation layer was then removed right after the exposure and before the resist developer, via wet etching. The final dipolar antennas are realized via deposition and liftoff of 10/80 nm Cr/Au metal layers. The optimized double-band antenna array was implemented by realizing and testing a set of samples with different array sizes (rod length, width, and spacing), then tuning the final design to precisely match the desired antenna resonances. Gold nanoantennas of different lengths in Fig. 2 were realized by applying a scaling factor to the original CAD 2D design of ± 10% and ± 5%.

Gold nanoantennas were functionalized according to Liu et al.[35]. Briefly, a 10 mM mixture of polyethylene glycol (PEG) molecules of two different molecular weights (3:1 molar ratio) was prepared in DPBS [113]. For this purpose, we used a short PEG chain (Methyl-PEG-Thiol MT(PEG)_4_, MW 200 kD, ThermoFisher Scientific) and a long PEG chain, namely biotinylated-polyethylene glycol-thiol (mPEG-Biotin, MW 1000 kD Nanocs). After PEG binding to the gold nanostructures, unbound PEG molecules were washed in DPBS, and the long PEG biotinylated chains were conjugated with Neutravidin (NeutrAvidin Biotin Binding, ThermoFisher). For this purpose, a 0.05 mg/mL neutravidin solution was prepared in DPBS [114, 115]. The surface of the device was further functionalized for exosome immunocapture using a 0.05 mg/mL Anti-CD63 biotinylated antibody [Anti-CD63 (MEM-259) BIOTIN ab134331, abcam] solution in DPBS and exploiting the high affinity of the multiple neutravidin binding sites for biotin.

Before measurements, the patterned CaF_2_ widow was arranged in a 3D-printed wet sample holder fabricated as follows. A 3D CAD of the device, consisting of three different components, was created with Rhinoceros®, then exported as a stl file to CURA software which created the G-Code script containing the commands to guide the printing process. The fabrication was realized by Fusion Deposition Modelling (FDM) employing an Ultimaker S3 printer and a Ultimaker Black Tough PLA filament (section of 2.85 mm). The filament was extruded at 210 °C, using a 0.25 mm diameter nozzle, and deposited on a heated build plate (65 °C). The devices were printed at 25 mm/s speed, taking about 8 h for the realization of the three components. The structures were printed layer by layer, each one with a 0.1 mm thickness, a line width of 0.23 mm, and a grid infill pattern at a density of 70%.

### IR measurements

The IR spectroscopy data were acquired with a vacuum Fourier-transform (FT) spectrometer (IFS66v by Bruker) with a KBr beamsplitter, coupled to a reflective-objective microscope (Hyperion by Bruker) equipped with a liquid nitrogen-cooled MCT detector (by Infrared Associates). The plasmonic-microfluidic device was installed onto the motorized stage of the microscope, with the focal plane of the top reflective objective positioned at the antenna plane. A square knife-edge aperture of 80 × 80 was selected and centered onto the given antenna array by visual inspection with the same microscope. The same aperture was positioned onto a flat gold layer to acquire reference spectra when needed, mostly to compensate for variations in the atmospheric CO_2_ and H_2_O content. The spectral resolution was set to 2 cm^−1^ and the interferogram sampling speed was 40 kHz. 512 interferograms were acquired in approximately 2 min and co-averaged before FT. A lithographic wire-grid polarizer (by Specac) on a KRS-5 (thalium bromoiodide) substrate was mounted inside the vacuum chamber of the FTIR spectrometer and remotely controlled so as to select the linear polarization of interest (almost everywhere, electric field co-polarized to the antenna axis). The IR nanospectroscopy data were acquired with a photothermal expansion nanospectrometer (NanoIR2 by Anasys-Bruker) equipped with a tunable quantum cascade laser (MIRcat xB by Daylight Solutions) and gold-coated scanning probes (by Anasys-Bruker). The solution with small extracellular vesicles was spotted on ultraflat gold substrates (by Platypus technologies) to achieve plasmonic field enhancement between probe tip and substrate. The reflectance and absorbance spectra were analyzed with the software IGOR PRO (by Wavemetrics).

### Cell culture

The human colorectal adenocarcinoma HT29 cell and Caco2 cell lines were cultured in DMEM High Glucose w/o Sodium Pyruvate (Euroclone- Pero, MI, Italy) supplemented with 10% (v/v) FBS (Euroclone), penicillin (100 IU/mL, Euroclone), streptomycin (100 µg/mL, Euroclone) l-glutamine (2 mM, Euroclone) and with and sodium pyruvate (1 mM, Euroclone) in a 5% CO_2_ humidified environment at 37 °C [83].

To isolate the small extracellular vesicles, both HT29 and Caco2 cells were seeded at 20.000 cells/cm^2^ in T-75 flasks.

For HT29 cells at 70–80% confluence, the cells were washed twice with DPBS without calcium chloride and magnesium chloride (DPBS 1X, Sigma-Aldrich, Germany), and DMEM was replaced with DMEM supplemented with 10% (v/v) FBS depleted of exosome and cultured for 24 h after the media change. While for Caco2 cells, 24 h after seeding the medium was changed to produce control cells and cells treated with 10 mM NAC according to our previous works [54, 59]. 72 h after treatment, the medium of both control and treated Caco2 cells, after washing with DPBS, was changed to DMEM with DMEM supplemented with 10% (v/v) FBS depleted of exosome and cultured for a further 24 h.

### Extracellular vesicles precipitation

The precipitation of EVs from cells’ media, for HT29 as well as for control and treated Caco2 cells, has been performed using the ExoQuick-TC precipitation kit (System Biosciences, CA, USA). We collected the supernatants 24 h after the media substitution with DMEM supplemented with FBS exosome-depleted. It was centrifuged for discharging dead cells, and cell debris with the appropriate g-force, and then 10 mL of supernatants were mixed with 2 mL of ExoQuick and incubated for 24 h at 4 °C. The mixtures were centrifuged at 1500*g* for 30 min, and then the supernatants were removed and re-centrifuged at 1500*g* for 5 min again. The EVs pellets were re-suspended in 500 µL of DPBS. Extracellular vesicles’ size distribution from HT-29 has been evaluated using Nanoparticle Tracking Analysis, Nanosight NS300 (NanoSight, UK), by dissolving the EVs pellet in a PBS solution (with a final concentration of about 10^10^ particles/ml).

The morphology of isolated EV was studied with AFM. For this purpose, freshly extracted small extracellular vesicles were diluted in de-ionized water (1:100), and a drop was deposited on a mica sheet (Ted 172 Pella, Inc. Redding, CA) at room temperature. AFM imaging was obtained in tapping mode in a dry environment using Nanowizard II (JPK Instrument, Germany) coupled with an optical microscope (Axio Observer, Carl Zeiss). Silicon cantilevers with a nominal spring constant of 7 N/m, tip radius of 8 nm, and a half conical angle of 40° was used (XSC11/NoAl-MikroMasch). EV biomechanics has been measured according to Vorselen et al. [116].

We also characterized the EVs morphology and size by Transmission Electron Microscopy using a TEM Libra120 (Zeiss, Germany). A drop of the EVs solution (about 10–20 μL) was covered with a copper mesh of 2 mm in diameter and then stained with a 10 μL drop of 2.5% Phosphotungstic Acid Solution (PTA), according to the protocol reported in Romanò et al. [83]. After the negative staining, the copper mesh was washed three times with deionized water at 37 °C. Thus, the sample was dried for a few minutes at 25 °C and then placed under TEM for image acquisition.

For immunoblotting, HT-29 cells and EVs pellets were suspended in RIPA lysis buffer following the protocol by Arteaga-Blanco et al. [117]. Protein concentration was measured using BCA Protein Assay Kit (Quantum protein cat.no. EMP014250; Euroclone). 40 µg of protein extract were separated using a 12% SDS/PAGE and then transferred to nitrocellulose membrane (Hybond, Amersham GE Healthcare). Membranes were then blocked, incubated with the CD63 (cat. no. ab8219; Abcam) or Cytochrome C antibody (cat. no. 556433; BD Biosciences). Primary antibodies were revealed with peroxidase-conjugated secondary antibody (cat. no. 1706516; BioRad) and subjected to enhanced chemiluminescence. ChemiDoc XRS + imager (Bio-Rad) was used for membrane, and exposure and images capture.

## Supplementary Information

**Additional file 1: Fig. S1.** Incident field enhancement at a distance of 25 nm from the unit cell of one of our patterned plasmonic surfaces, as computed with the Green Dyadic Method. **Fig. S2.** Enlarged detail of the near-field enhancement for the long nanoantenna as a function of the distance from the patterned surface in the range of 0-400 nm. **Fig. S3.** Representative SEM micrograph of HT29 cancer cells utilized for EVs precipitation from cell media. Scale bar 5 μm. **Fig. S4**. Western Blot (left) and NTA analysis (right) of the EVs extracted form Caco2 cacer cells. **Fig. S5.** NA redshift due to the interaction between EVs and the metasurface in the presence (blue filled dots ) and the absence ( open black dots) of Anti-CD63 functionalization. A schematic representation of the two hypothesized interaction models is reported in the top right and bottom right diagrams. **Fig. S6.** Representative FD curves acquired on EV particles captured on a gold functionalized surface (left). Topographical image of two different Nanoantennas, measured before gold functionalization and after EV immunocapture (right). A line profile with roughness is reported to show increased roughness.

## Data Availability

Data are available from the corresponding author upon reasonable requests.

## Abbreviations

AFM: Atomic force microscopy
ATR: Attenuated total reflectance
DLS: Dynamic light scattering
DMEM: Dulbecco’s modified eagle medium
DPBS: Dulbecco’s phosphate buffer saline
EBL: Electron beam lithography
EV: Extracellular vesicle
FTIR: Fourier-transform infrared spectroscopy
HCC: Hepatocellular carcinoma
NA: Nanoantenna
PDMS: Polydimethylsiloxane
PEG: Polyethylene glycol
PIR: Plasmonic internal reflection
PLA: Polylactic acid
PMMA: Poly(methyl methacrylate)
RI: Refractive Index
SEIRA: Surface-enhanced infrared absorption spectroscopy
SEM: Scanning electron microscopy
sem: Standard error of the mean
SERS: Surface-enhanced Raman spectroscopy
SPR: Surface plasmon resonance
TEM: Transmission electron microscopy
