# Supplementary material for: Antenna-enhanced mid-infrared detection of extracellular vesicles derived from human cancer cell cultures
Source: J Nanobiotechnology. 2022 Dec 13;20:530. doi: 10.1186/s12951-022-01693-2 (PMC9746222; doi:10.1186/s12951-022-01693-2)
Supplement: Supplementary file 1 — Additional file 1: Fig. S1. Incident field enhancement at a distance of 25 nm from the unit cell of one of our patterned plasmonic surfaces, as computed with the Green Dyadic Method. Fig. S2. Enlarged detail of the near-field enhancement for the long nanoantenna as a function of the distance from the patterned surface in the range of 0-400 nm. Fig. S3. Representative SEM micrograph of HT29 cancer cells utilized for EVs precipitation from cell media. Scale bar 5 μm. Fig. S4. Western Blot (left) and NTA analysis (right) of the EVs extracted form Caco2 cacer cells. Fig. S5. NA redshift due to the interaction between EVs and the metasurface in the presence (blue filled dots ) and the absence ( open black dots) of Anti-CD63 functionalization. A schematic representation of the two hypothesized interaction models is reported in the top right and bottom right diagrams. Fig. S6. Representative FD curves acquired on EV particles captured on a gold functionalized surface (left). Topographical image of two different Nanoantennas, measured before gold functionalization and after EV immunocapture (right). A line profile with roughness is reported to show increased roughness. [file 12951_2022_1693_MOESM1_ESM.docx]

# Supplementary Materials

**Electrodynamic Simulations of gold Nanoantennas and estimation of the minimum number of measured EV particles.**

In figure S1, we report a representative electrodynamic simulation of the unit cell of one of our patterned plasmonic surfaces reported in figure 2a. The near-field electromagnetic field enhancement is shown on the z color scale$\left( \frac{E^{2}}{E_{0}^{2}} \right)$.


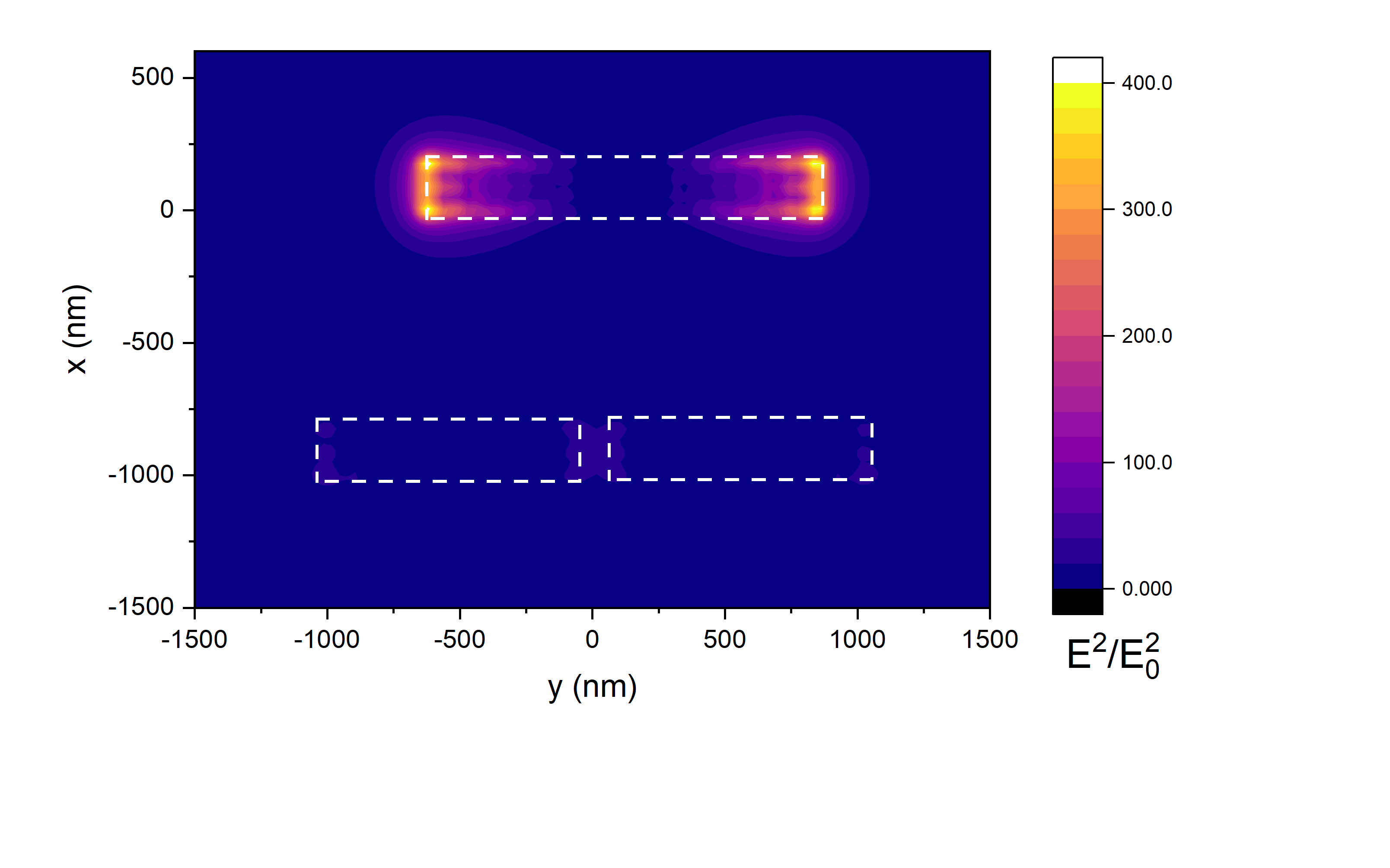


Figure S1: *Incident field enhancement at a distance of 25 nm from the unit cell of one of our patterned plasmonic surfaces, as computed with the* Green Dyadic Method

The simulation is performed in air with the Green dyadic Method according to ref 42, at normal incidence, for gold nanostructures deposited on a CaF_2_ substrate (n=1,43), and with an excitation length of 6100 nm (in the Amide I band). E^2^/E^2^_0_ is computed at a distance of 25 nm from the gold surface using an unpolarised incident electric field. As expected, for this incident wavelength, only the long nanoantennas appear to be excited, while the contribution of the two shortest ones is almost negligible. A linear scale is used on the z colour scale axis.

In figure S2, we show enlarged detail of the field enhancement for the long nanoantenna as a function of the distance from the patterned surface in the range of 0-400 nm. A logarithmic z scale was used to better visualize the field distribution, which undergoes large variation with the horizontal and vertical coordinates. At the nanostructure surface (z=0), we have a maximum field enhancement as high as ~1500 times the incident field. At approximately 400 nm, the maximum field enhancement is slightly above 1, therefore we have no more signal amplification. To visualize such a high variation in the field enhancement, we used different logarithmic colour scales at different distances from the surface.

Figure S2: Enlarged detail of the near-field enhancement for the long nanoantenna as a function of the distance from the patterned surface in the range of 0-400 nm.

We used the simulation in figure S2 in combination with literature data (Koliha et al., ref 52) to estimate the minimum number of measurable EVs. More specifically, from figure 3, we computed for our device an ideal protein sensitivity down to 10^-20^ moles. This estimation was performed on a 150kD molecule, which leads us to a mass sensitivity of approximately $1.5\cdot{10}^{-15}g$ of protein. Koliha et al. estimated a protein concentration per particle ranging from ${10}^{-16}g/EV$ to ${10}^{-14}g/EV$, studying different EV types [52]. For a conservative estimation, we used the lower bound of Koliha’s interval, which leads us to a minimum number of a few tens of EVs ($\frac{1.5\cdot{10}^{-15}g}{{10}^{-16}g/EV})$. Concerning this result, a caveat is necessary: the obtained sensitivity is largely overestimated, as the utilized protein sensitivity $\left( 1.5\cdot{10}^{-15}g \right)$was obtained for molecules located approximately on the surface of the nanostructures, where the field enhancement is maximum. On a contrary, our EV absorption signals come from the proteins within EVs, which are located at larger distances above the surface. Our NTA measurements (figure 4) show a modal EV diameter of approximately 100 nm and the distribution shape appears strongly right-skewed. Assuming a uniform protein distribution within the EV cargo, we can estimate an average protein distance from the surface, which ranges between 50 to 100 nm. In this z range, field enhancement decreases significantly compared to the nanostructure surface. The decrease in the amplification factor can be estimated from the scatter plot in figure S2. Compared to the NA surface (z=0), we observed a decrease in the field amplification ranging from approximately 10 to 30 between 50 to 100 nm above NAs, which leads to an increase in the minimum number of measured EVs up to several hundreds of particles.

We believe that this estimation provides an extremely interesting number as it is an intermediate value between nanoscopy ( which can reasonably take into account from a few to a few tens of particles with large experimental times) and bulk methods such as ATR-FTIR, which requires a significantly larger amount of the particles. In our opinion, the possibility to obtain an informative signal by using such a small number of particles is instrumental for the development of novel liquid biopsy methods based on the analysis of complex biofluids. More specifically, cancer-derived EVs are likely a minority component in complex media, such as serum. If the number of EVs of such a minor component is too low, bulk methods would not be effective in terms of signal-to-noise ratio (SNR). On the contrary, the synergistic effect of immunocapture and SEIRA enhancement could, in principle, provide the required SNR to measure accurate average spectral features on small EV subpopulations.

***Additional cellular and EV characterizations***


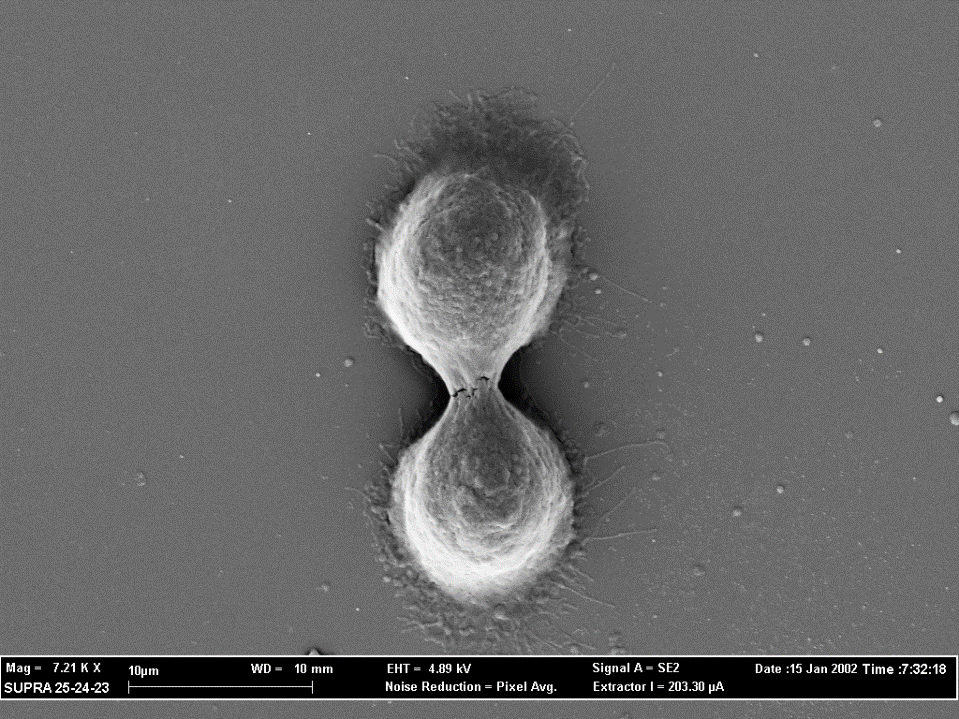


Figure S3: representative SEM micrograph of HT29 cancer cells utilized for EVs precipitation from cell media. Scale bar 5 μm

In figure S3, we show a representative SEM image of HT-29 cancer cells cultured in EV-free DMEM. The cells show the expected morphology, and appear to be properly adherent to the Petri surface, as confirmed by the presence of a large amount of rod-like protrusions filled with bundles of parallel actin filaments, most frequently termed filopodia. Cell viability was also verified obtained a percentage of alive cells of 95%.


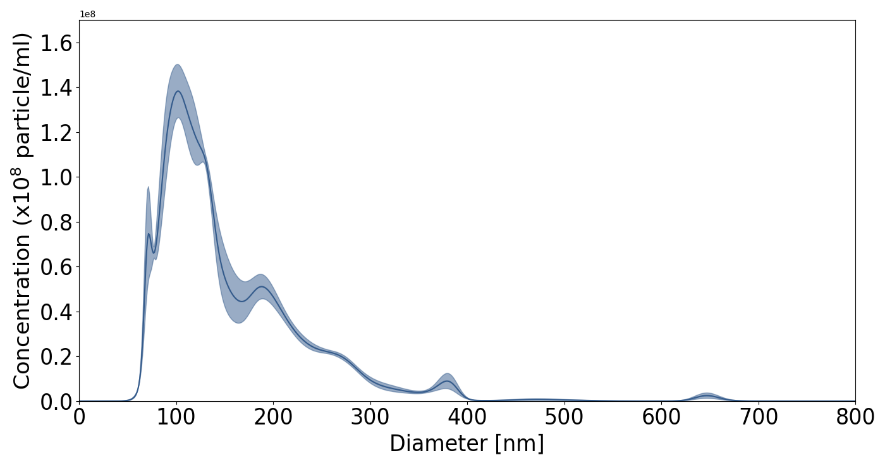

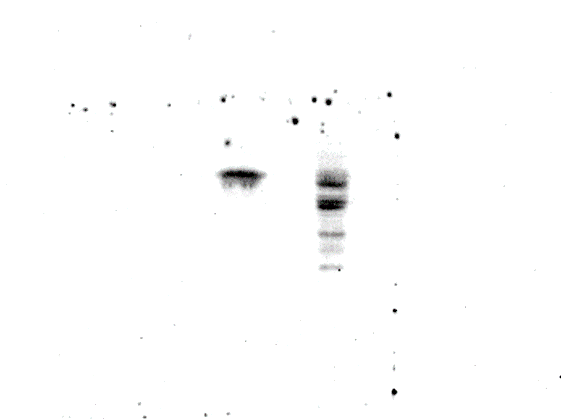

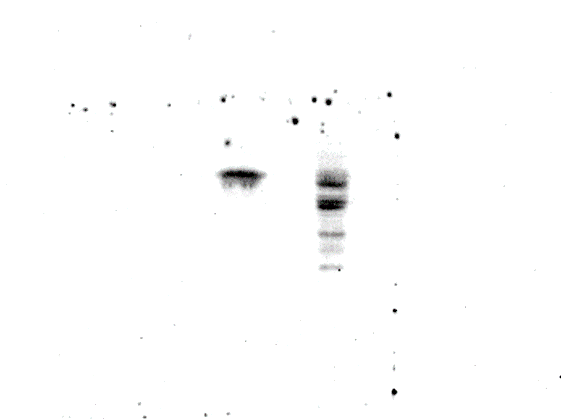


**CD-63**

(70-30kDa)

**Cyto C**

(15 kDa)


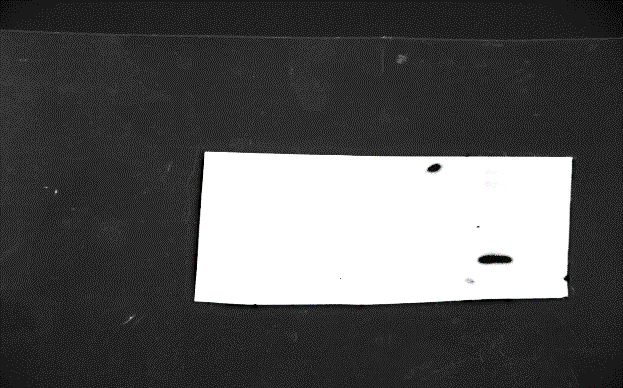


**CACO-2**

Cellss

EVs

Figure S4 western Blot (left) and NTA analysis (right) of the EVs extracted form Caco2 cacer cells.

In figure S4 (left) we report a Western blot analysis of EV markers (CD63) and non-Ev markers (Cytochrome C) in Caco2 cell-derived EVs and in the whole-cell lysate. A clear increase in the expression of CD63 is observed in the EV sample compared to the whole cell lysate. As expected, the negative marker cytochrome C is detectable only in the whole cell lysate while is absent in the Caco2-derived EV samples. In the right panel, we report the corresponding NTA analysis, which shows a multimodal distribution with the main peak at approximately 100 nm.

***Additional experimental evidence on the role of Anti-CD63***


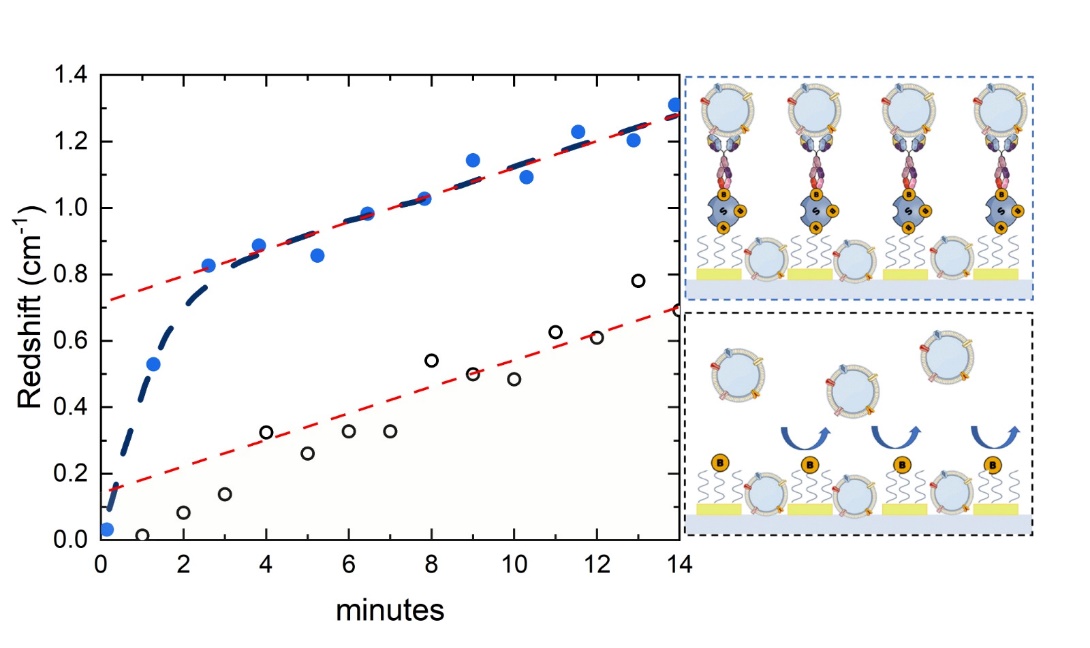


Figure S5: NA redshift due to the interaction between EVs and the metasurface in the presence (blue filled dots ) and the absence ( open black dots) of Anti-CD63 functionalization. A schematic representation of the two hypothesized interaction models is reported in the top right and bottom right diagrams.

As discussed in the main manuscript, our functionalization strategy is based on the use of the following sequence of biomolecules: biotinylated-PEG, Neutravidin, and biotinylated Anti-CD63, which is then used for EV immunocapture. In figure S5, we provide additional information on the role of AntiCD63 functionalization in modulating the interaction between EVs and the metasurface. More specifically, we monitored the EV interaction with our device in the absence of Anti-CD63 and we compared it with our results in figure 5d. A significant decrease in the Antenna redshift is measured in the absence of Anti-CD63 (open black dots) compared to the fully functionalized device (blue-filled dots), confirming the effectiveness of the functionalization with anti-CD63. Additionally, different dynamical behaviors can be observed in the two experimental setups. In the fully functionalized device, we observe an abrupt increase in the redshift response in the first few minutes, followed by a slower linear increase highlighted with a red dashed line. Notably, in the absence of Anti-CD63, the first abrupt increase is not observed and a mild increase in the redshift is measured. Very interestingly, this trend highly resembles the linear trend in the fully functionalized device, as graphically highlighted by a vertical shift of the aforementioned red-dashed line. These results are consistent with the coexistence of two phenomena in the fully functionalized device: i) a rapidly occurring and specific interaction between EVs and the metasurface mediated by immunocapture, which is likely to saturate because of the decrease in the available binding sites; ii) an unspecific and minority interaction of the analytes with the free CaF_2_ surface between the nanostructures. Conversely, in the absence of AntiCD63, we can observe only the second phenomenon, as EVs cannot bind the gold nanoantennas which are shielded by the unwanted interaction because of the peg mixture.

***AFM study of EV captured from flat and nanostructured functionalized gold surfaces***

In this paragraph, we further verified the effectiveness of our functionalization strategy for EV immunocapture on flat gold (left panel) and our nanostructured surface (right panel). AFM biomechanics of extracellular vesicles is currently a topic of high interest in the literature, with several papers on the subject. In their excellent methodological paper, Vorselen et al. [131] described in-depth the typical biomechanical response of EVs, as measured with AFM force-distance (FD) curves, highlighting the presence of several key hallmarks on a flat surface. Here, a flat gold surface was functionalized by using the same protocol exploited for the device treatment. AFM in the force spectroscopy mode was used to detect EVs on the surface and to acquire FD curves. Two representative FD curves are shown in figure S6. A qualitative analysis of the reported FD curves shows several hallmarks, which are compatible with the presence of EVs according to [131], i) a smooth indentation between the contact point and the EV height (approximately between the contact point and 20 nm); ii) an increase in reaction force when the two bilayers are pressed together followed by the presence of two discontinuities indicating 1st (the zero point in our x-axes) and 2nd bilayer rupture. The region corresponding to the lipid bilayer is highlighted in cyan; iii) an increase of the reaction force as a consequence of the interaction with the substrate. Ultimately, we report two topographical AFM images of two different nanoantennas belonging to two different devices patterned on the same CaF_2_ substrate, before gold functionalization, and after EV immunocapture. The Nanoantenna’s morphology appears to be significantly altered after the functionalization process, albeit we are not able to assess whether this modification is due to EVs or other molecules absorbed on the surface.


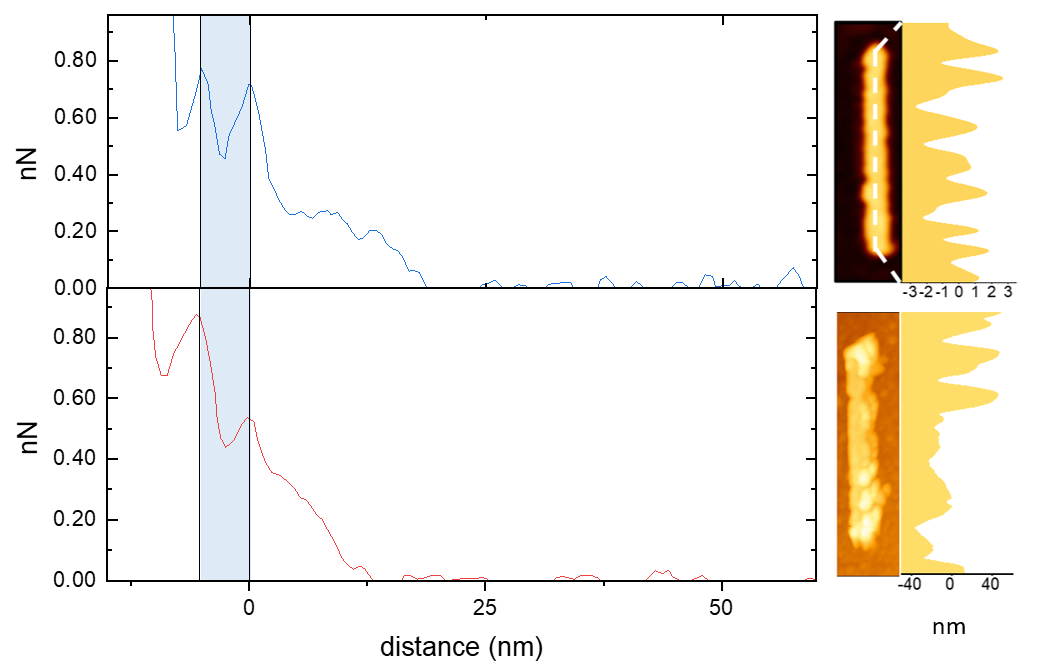


Figure S6: representative FD curves acquired on EV particles captured on a gold functionalized surface (left). Topographical image of two different Nanoantennas, measured before gold functionalization and after EV immunocapture (right). A line profile with roughness is reported to show increased roughness.
